# Integrated network analysis and metabolomics reveal the molecular mechanism of Yinchen Sini decoction in CCl_4_-induced acute liver injury

**DOI:** 10.3389/fphar.2023.1221046

**Published:** 2023-09-25

**Authors:** Weiwei Zheng, Chao Shi, Yao Meng, Jian Peng, Yongfei Zhou, Tong Pan, Ke Ning, Qiuhong Xie, Hongyu Xiang

**Affiliations:** ^1^ Key Laboratory for Molecular Enzymology and Engineering of Ministry of Education, School of Life Sciences, Jilin University, Changchun, Jilin, China; ^2^ National Engineering Laboratory for AIDS Vaccine, School of Life Sciences, Jilin University, Changchun, Jilin, China; ^3^ Institute of Changbai Mountain Resource and Health, Jilin University, Fusong, Jilin, China

**Keywords:** Yinchen Sini decoction, acute liver injury, network analysis, 1 H-NMR, PI3K-AKT

## Abstract

**Objective:** Yinchen Sini decoction (YCSND), a traditional Chinese medicine (TCM) formula, plays a crucial role in the treatment of liver disease. However, the bioactive constituents and pharmacological mechanisms of action remain unclear. The present study aimed to reveal the molecular mechanism of YCSND in the treatment of acute liver injury (ALI) using integrated network analysis and metabolomics.

**Methods:** Ultra-high-performance liquid chromatography coupled with Q-Exactive focus mass spectrum (UHPLC-QE-MS) was utilized to identify metabolites in YCSND, and high-performance liquid chromatography (HPLC) was applied to evaluate the quality of four botanical drugs in YCSND. Cell damage and ALI models in mice were established using CCl_4_. ^1^H-NMR metabolomics approach, along with histopathological observation using hematoxylin and eosin (H&E), biochemical measurements, and reverse transcription quantitative real-time PCR (RT-qPCR), was applied to evaluate the effect of YCSND on CCl_4_- induced ALI. Network analysis was conducted to predict the potential targets of YCSND in ALI.

**Result:** Our results showed that 89 metabolites in YCSND were identified using UHPLC-QE-MS. YCSND protected against ALI by reducing the levels of alanine aminotransferase (ALT), aspartate aminotransferase (AST), and malondialdehyde (MDA) contents and increasing those of superoxide dismutase (SOD), and glutathione (GSH) both in vivo and in vitro. The ^1^H-NMRmetabolic pattern revealed that YCSND reversed CCl_4_-induced metabolic abnormalities in the liver. Additionally, the Kyoto Encyclopedia of Genes and Genome (KEGG) pathway enrichment analysis identified five pathways related to liver injury, including the PI3K-AKT, MAPK, HIF-1, apoptosis, and TNF signaling pathways. Moreover, RT-qPCR showed YCSND regulated the inflammatory response (*Tlr4*, *Il6*, *Tnfα*, *Nfκb1*, *Ptgs2*, and *Mmp9*) and apoptosis (*Bcl2*, *Caspase3*, *Bax*, and *Mapk3*), and inhibited PI3K-AKT signaling pathway (*Pi3k* and *Akt1*). Combined network analysis and metabolomics showed a link between the key targets (*Tlr4*, *Ptgs2*, and *Mmp9*) and vital metabolites (choline, xanthine, lactate, and 3-hydroxybutyric acid) of YCSND in ALI.

**Conclusion:** Overall, the results contribute to the understanding of the therapeutic effects of YCSND on ALI, and indicate that the integrated network analysis and metabolomics could be a powerful strategy to reveal the pharmacological effects of TCM.

## 1 Introduction

Acute liver injury (ALI) is a reversible wound-healing process caused by several xenobiotic and toxic metabolites in the liver, particularly chemical toxins, drugs, alcohol, and viruses (Ren et al., 2019). CCl_4_ is a powerful hepatoxic and nephrotoxic agent that is widely used to induce hepatotoxicity in experimental animals and to establish models of acute/chronic liver injury, hepatic fibrosis/cirrhosis, hepatic failure and nephrotoxicity (Unsal et al., 2021). CCl_4_-induced ALI possesses similar molecular mechanisms with human ALI, making it a suitable model for ALI research. CCl_4_ is metabolized by hepatic cytochrome P450 enzymes (CYP450), producing reactive oxygen species (ROS), and excessive ROS production can induce oxidative stress, inflammatory response, and hepatocyte apoptosis, all of which play a pivotal role in ALI pathogenesis. (Xie et al., 2018). In the absence of effective treatment, ALI can evolve into more severe liver diseases, such as chronic liver injury, liver fibrosis, and even irreversible liver cirrhosis and cancer. Currently, there is no safe and effective therapeutic drug for ALI owning to its multiple pathogenic factors and complicated pathological processes. In China, some common hepatoprotective drugs used to treat ALI include silymarin and magnesium isoglycyrrhizinate (Li M. et al., 2022; Liu et al., 2022). However, the use of these drugs is associated with several issues, including limited therapeutic efficacy and severe side effects (Xie et al., 2018; Ren et al., 2019), indicating the need to develop more effective and safer drugs for ALI treatment.

Traditional Chinese medicine (TCM) formulas are commonly used to treat ALI, with the advantages of fewer side effects and multiple metabolites with several targets (Zhang et al., 2018). Several TCM formulas have been successfully used to treat CCl_4_-induced ALI (Lee et al., 2020; Wei et al., 2021; Zhao Q. et al., 2022). Yinchen Sini decoction (YCSND), a TCM formula from the Song dynasty, is composed of four Chinese botanical drugs, *Artemisia capillaris* Thunb. [Asteraceae; Artemisiae scopariae herba], *Aconitum carmichaelii* Debeaux [Ranunculaceae, Aconiti lateralis radix praeparata], *Zingiber officinale* Roscoe [Zingiberaceae, Zingiberis rhizoma], and honey-fried *Glycyrrhiza glabra* L. [Fabaceae, Glycyrrhizae radix et rhizoma praeparata cum melle]. Among the four botanical drugs, *Artemisia capillaris* Thunb. [Asteraceae; Artemisiae scopariae herba] and *Aconitum carmichaelii* Debeaux [Ranunculaceae, Aconiti lateralis radix praeparata] possess antioxidant, anti-inflammatory, and anti-apoptotic effects; *Zingiber officinale* Roscoe [Zingiberaceae, Zingiberis rhizoma] and honey-fried *Glycyrrhiza glabra* L. [Fabaceae, Glycyrrhizae radix et rhizoma praeparata cum melle] mainly play harmonic roles in detoxifying *Aconitum carmichaelii* Debeaux [Ranunculaceae, Aconiti lateralis radix praeparata], and these metabolites of YCSND synergistically exert positive effects in the body (Wang M. F. et al., 2021; Wang Y. et al., 2021; Huang et al., 2021). Recent pharmacological studies have shown that YCSND has hepatoprotective properties, and some metabolites occurring in YCSND, including scoparone (Hsueh et al., 2021), glycyrrhizic acid (Huo et al., 2020), and chlorogenic acid (Merzouk et al., 2017) have been reported to exert hepatoprotective effects. YCSND has been clinically used to treat hepatobiliary disease (Wang et al., 2008; Chen et al., 2019) and was effective against hepatic inflammation in a mouse model of intrahepatic cholestasis (Wang Q. et al., 2020). However, studies on the effect of YCSND in CCl_4_-induced liver injury are limited. For instance, Li et al. (2016a; 2016b) revealed that combined treatment with YCSND and *Conioselinum anthriscoides* [Apiaceae; Ligusticum Chuanxiong rhizoma] may potentially prevent CCl_4_-induced chronic liver injury by significantly reducing alanine aminotransferase (ALT) and aspartate aminotransferase (AST) levels, *Smad3* and *Smad7* mRNA expression, and collagen I and III expressions in the liver. However, the effectiveness of only YCSND in ALI treatment, and its molecular mechanism are yet to be elucidated.

Traditional network analysis is typically used to predict potential targets and molecular mechanisms of TCM in disease treatment, using data mining and scientific computing techniques based on public databases (Yang et al., 2023). Network analysis can reveal the patterns and rules that govern the associations between drugs and diseases, enabling efficient screening of potential drug targets and pathways with therapeutic effects. However, the accuracy and reliability of the predictions may be skewed owing to limitations in the quality of the database information, such as errors, noise, and biases (Li Z. et al., 2022). Moverover, the composition of a TCM formula may not be a simple superposition of individual plant-derived metabolites. During the processing of TCM formulas, botanical drugs may exhibit synergistic, cooperative, or inhibitory effects, potentially accompanied by changes in their metabolites (Li Z. et al., 2022). Due to these complexities, the conventional network analysis approach, which relies on public databases to screen plant-derived ingredients, appears to be unreliable for studying TCM formulations. Network predictions using actual constituents of plant-derived formulations appear to be more dependable than conventional databases. Therefore, it is urgent and necessary to develop a new method of network analysis for the study of TCM metabolites to improve the accuracy of network prediction.

^1^H-NMR technology is a beneficial tool for analyzing metabolites and has several advantages, such as reliable results, nondestructive sampling, and minimal sample preparation (Borém et al., 2023). Integrated of network analysis and metabolomics has unique advantages in the study of TCM formulas, as it can reveal the intricate mechanisms of action within the body from multiple perspectives, including metabolites of botanical drug, targets, pathways, and metabolites *in vivo* (Zhao Q. et al., 2022). Network analysis and metabolomics have successfully been applied to uncover the mechanisms of several TCM formulas in liver disease treatment (Yu et al., 2019; Wang et al., 2022; Yang et al., 2023).

**FIGURE 1:**
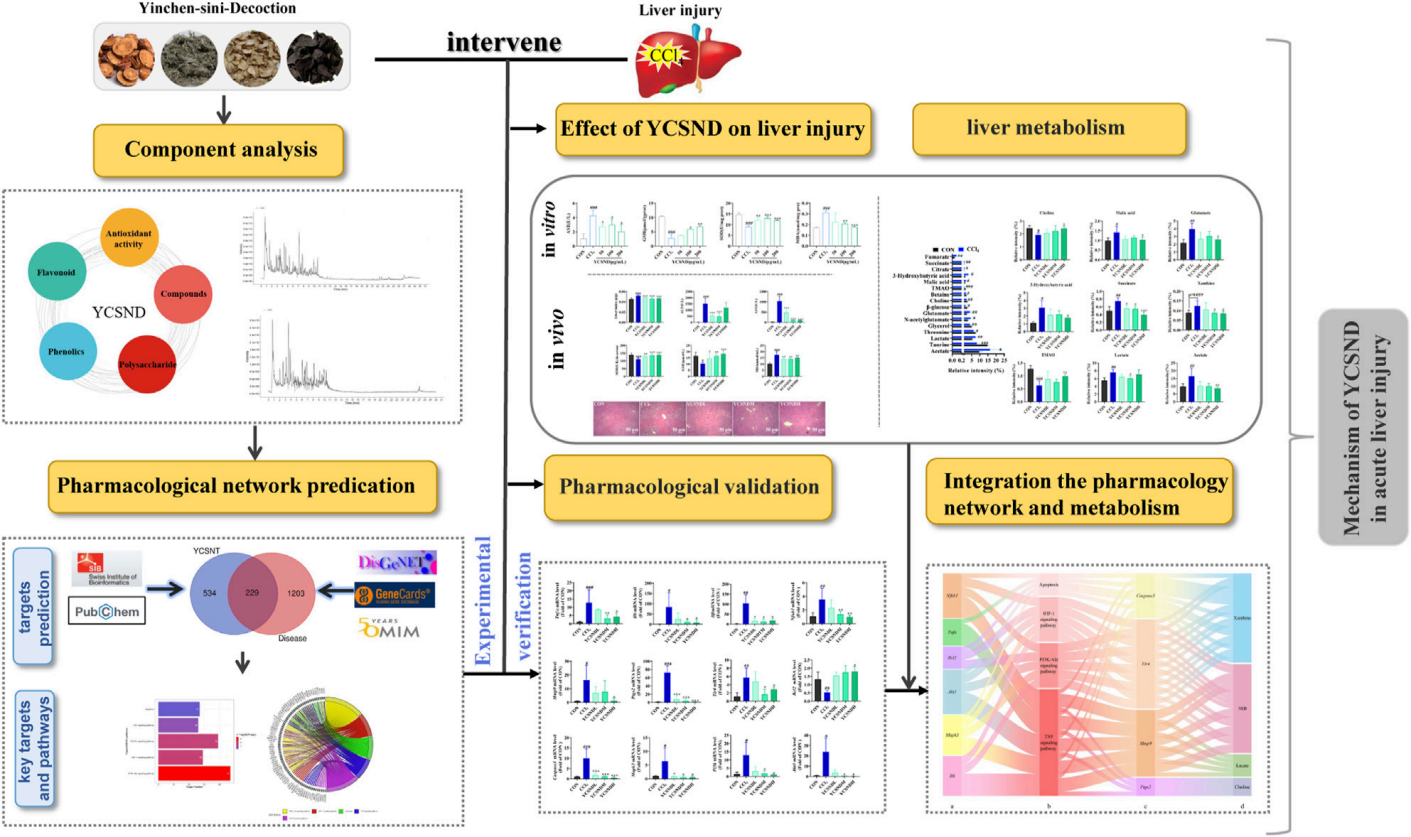
Graphic abstract.

In this study, a novel network prediction method based on the actual metabolites of YCSND was employed. This approach improves upon traditional blind screening by enhancing the accuracy of network predictions and it also enables faster and more precise identification of effective targets and pathways facilitating a more efficient exploration of underlying mechanisms. Additionally, the predicted targets and pathways were validated using reverse transcription quantitative real-time PCR (RT-qPCR). Moreover, network predictions and metabolomics were integrated to elucidate the molecular mechanisms of YCSND in liver injury from multiple perspectives. The present study serves as a theoretical basis for the therapeutic use of YCSND in liver diseases, offering novel insights into the study of TCM formulations. The graphic abstract of this study is shown in Figure 1.

## 2 Materials and methods

### 2.1 Preparation of YCSND

Botanical drugs for YCSND, including *Artemisia capillaris* Thunb. [Asteraceae; Artemisiae scopariae herba], *Aconitum carmichaelii* Debeaux [Ranunculaceae, Aconiti lateralis radix praeparata], *Zingiber officinale* Roscoe [Zingiberaceae, Zingiberis rhizoma], and honey-fried *Glycyrrhiza glabra* L. [Fabaceae, Glycyrrhizae radix et rhizoma praeparata cum melle] were purchased from the drug department of Yunling grass bouquet (Yunnan, China) and were authenticated by Prof. Yuguang Chen (Changchun traditional Chinese medicine hospital). YCSND was prepared using 10 g each of *Artemisia capillaris* Thunb. [Asteraceae; Artemisiae scopariae herba], *Aconitum carmichaelii* Debeaux [Ranunculaceae, Aconiti lateralis radix praeparata], *Zingiber officinale* Roscoe [Zingiberaceae, Zingiberis rhizoma], and honey-fried *Glycyrrhiza glabra* L. [Fabaceae, Glycyrrhizae radix et rhizoma praeparata cum melle]. Quality control for the four botanical drugs for YCSND met the standards of the Chinese Pharmacopoeia (Table 1). The botanical drug mixture (40 g) was soaked and decocted with 400 mL of distilled water for 45 min. The first liquid was obtained by centrifuging at 4,000 rpm for 10 min, and the residues were sequentially extracted twice with an equal volume of water for another 45 min. The supernatants were then mixed and concentrated in a rotary evaporator. Finally, the concentrated liquid was stored at −80°C for 24 h and dried in a freeze dryer for another 36 h, and the freeze-dried YCSND powder (with an extraction rate of 35.6%) was stored at 4°C for further experiments.

**TABLE 1:** Contents of the main identified metabolites in YCSND extract.

Plant Name	Metabolites	Retention time (min)	Wavelength (nm)	Contents (mg/g)
*Artemisia capillaris* Thunb. [Asteraceae; Artemisiae scopariae herba]	chlorogenic acid	11.442	325	7.602
*Zingiber officinale* Roscoe [Zingiberaceae, Zingiberis rhizoma]	6-gingerol	18.749	230	6.520
*Aconitum carmichaelii* Debeaux [Ranunculaceae, Aconiti lateralis radix praeparata]	benzoylmesaconine	19.154	235	0.089
	benzoylaconine	25.753		0.005
	benzoylhypacoitine	55.053		0.147
honey-fried *Glycyrrhiza glabra* L. [Fabaceae, Glycyrrhizae radix et rhizoma praeparata cum melle]	liquiritin	8.561	254	5.311
	glycyrrhizic acid	34.542		25.348
YCSND	chlorogenic acid	11.442	325	2.029
	scoparone	40.563		1.441
	caffeic acid	14.861		0.092
	isochlorogenic acid B	40.563		0.023
	6-gingerol	18.749	230	1.416
	6-shogaol	31.227		1.860
	liquiritin	8.561	325	1.024
	glycyrrhizic acid	34.542		7.333
	benzoylmesaconine	19.154	235	0.051
	benzoylaconine	25.753		0.005
	benzoylhypacoitine	55.053		0.052
	neoaconitine	39.922		0.007
	hypaconitine	45.690		0.001
	aconine	50.180		No detected

### 2.2 Metabolite analysis of YCSND

Total phenolic, total flavonoid and polysaccharide contents were determined using previously described methods (Sahayarayan et al., 2020; Wu et al., 2021; Mogadem et al., 2022). 2,2-diphenyl-1-picrylhydrazyl radical (DPPH), 2,2′-azino-bis (3-ethylbenzothiazoline-6-sulphonic acid) radical (ABTS), superoxide radical (O_2_^−^), and hydroxyl radical (·OH) scavenging activities were determined using previously described methods (Siddhuraju and Manian, 2007), Vitamin C as the positive control. Metabolite analysis of YCSND was performed using ultra-high-performance liquid chromatography coupled with Q-Exactive focus mass spectrum (UHPLC-QE-MS) at Biotree Biomedical Technology CO., Ltd (Shanghai, China). High-performance liquid chromatography (HPLC) was used to analyze for chemical fingerprints and quantification of metabolites of YCSND. Detailed procedures of UHPLC-QE-MS and HPLC are described in the Supplementary Materials (Supplementary Table S1).

### 2.3 Cell pretreatment

HepG2 cells in the exponential growth phase were seeded into cell plates for 24 h, followed by treatment with YCSND (50, 100, and 200 μg/mL) for another 12 h. Cells in the normal and model groups were treated with equal volumes of DMEM instead of YCSND. Subsequently, the cells were treated with CCl_4_ (final concentration 0.05%, diluted with DMSO) for 12 h, except those in the normal group. Finally, the prepared cells were used for subsequent analyses.

### 2.4 Cell viability assay

Cell viability was determined using the MTT assay method (Bilkan et al., 2022). Briefly, MTT (5 mg/mL, 20 μL/per well) was added to the cells and incubated at 37°C for 4 h. Subsequently, the solutions were removed, DMSO (100 μL/well) was added to the wells, and absorbance was measured at 490 nm using a microplate reader (BioTek, USA).

### 2.5 Animal experiment design

Male Kunming mice (4-5 weeks old; 18–22 g) were purchased from Liaoning Changsheng Biotechnology. All animal experimentation procedures were approved by the Animal Care and Use Committee of Jinlin University (approval no.: 2021SY0715). The mice were housed under standard conditions at 23°C ± 2°C in a 12-h light/dark cycle and fed standard mice chow and water. After adaptive feeding for 7 d, the mice were randomly assigned to five groups (six mice per group): control (CON), CCl_4_-treated (CCl_4_), low-dose YCSND (YCSNDL), medium-dose YCSND (YCSNDM), and high-dose YCSND groups (YCSNDH). Mice in the CON and CCl_4_ groups were treated with physiological saline, whereas those in the three YCSND groups were treated with 400, 800, and 1,600 mg/kg body weight of YCSND powders per day, respectively. According to the description of the body surface area normalization method between humans and animals (Reagan-Shaw et al., 2008), the dose of humans was converted into the dose of mice: 40 g raw drugs of YCSND per day for a 70 kg person is equivalent to 1,600 mg/kg YCNSD extract as the high dose. The intragastric administration was continued for 21 d. All mice were intraperitoneally injected with 0.3% CCl_4_ (v/v, 10 mL/kg, diluted in olive oil) 2 h after the last administration, except for mice in the CON group, which were injected with an equal volume of olive oil. After 24 h, all mice were euthanized by carbon dioxide inhalation (Supplementary Figure S1), and liver samples and serum were collected for further analyses.

### 2.6 Determination of physiological index

ALT and AST levels in cell culture supernatants and serum were determined using standardized kits (Jiancheng, Nanjing, China), according to the manufacturer’s instructions. Superoxide dismutase (SOD) and glutathione (GSH) activities and malondialdehyde (MDA) contents of HepG2 cells and serum were determined using standardized kits (Jiancheng, Nanjing, China), according to the manufacturer’s instructions. Cell protein contents were measured using a bicinchoninic acid (BCA) protein assay kit (Bioss, Beijing, China).

### 2.7 Liver histopathology assay

A small piece of the cut right lobe of the liver was fixed in a 10% formalin solution, dehydrated, made transparent, paraffin-embedded, and cut into 2-mm thick sections. Thereafter, the liver tissue was stained with hematoxylin and eosin (H&E) and viewed using a light microscope (Shrestha et al., 2016).

### 2.8 RNA extraction and RT-qPCR

Total RNA extraction of the liver tissue, cDNA synthesis, and qPCR were performed according to previously described procedures (Wang Y. et al., 2020) RT-qPCR was performed using the RealStar Green Fast Mixture (Genstar, Beijing, China). Glyceraldehyde-3-phosphate dehydrogenase (*Gapdh*) was selected as the internal control for RT-qPCR and subsequent normalization. The relative expression levels of target genes were calculated using the comparative CT method (2^−ΔΔCT^). The primer sequences of the genes are listed in Supplementary Table S2.

### 2.9 ^1^H-NMR metabonomic analysis of the liver

Sample preparation for ^1^H-NMR was performed according to previously described methods with slight modifications (Liu et al., 2021). Briefly, approximately 200 mg of frozen liver samples were homogenized with 600 μL of methanol and ultra-water mixture (v/v, the ratio of 2:1) for 30 s, and the treated samples were centrifuged at 12,000 rpm for 10 min at 4°C. Thereafter, the above procedure was repeated twice. Cell supernatant from the three extraction were mixed in a 2 mL tube and methanol was removed by evaporation. Subsequently, the treated samples were resuspended and vortexed uniformly in 750 μL of 0.1 M phosphate buffer (pH = 7.4) with 0.01% TSP and 10% D_2_O. To remove insoluble ingredients, the extracts were centrifuged at 12,000 rpm for 10 min at 4°C. Finally, 550 μL of the supernatant was transferred to an NMR tube for ^1^H NMR detection. ^1^H-NMR detection and spectral processing were performed according to previously described methods (Ruiz-Rodado et al., 2016). Orthogonal partial least squares-discriminant analysis (OPLS-DA) was applied to multivariate pattern recognition analysis using SIMCA (version 14.0, UMETRICS, Umeå, Sweden). Metabolites with variable importance for projection (VIP) > 1 and *p* < 0.05 were identified as differentially expressed metabolites between the two groups based on *t*-test. Metabolites were assigned using the Human Metabolome Database (http://www.hmdb.ca), and metabolomics pathway analysis was performed using MetaboAnalyst (https://www.metaboanalyst.ca).

### 2.10 Network analysis

Network analysis was used to predict the potential molecular mechanism of YCSND in liver injury. The targets of the active metabolites in YCSND were determined following previously described method (Guo et al., 2021). The candidate targets of ALI were screened by searching the keywords “liver injury” in the OMIM database (https://www.omim.org/), DisGeNET database (https://www.disgenet.org/), and GeneCard database (https://www.genecards.org/). The species “*Homo sapiens*” was selected, and the target gene information related to liver injury was collected and integrated. Venn diagram was used to obtain intersecting targets from the active ingredient targets of YCSND and the liver-injury targets. An intersecting target network between the metabolites of YCSND and targets was constructed using Cytoscape 3.8.2. Gene ontology (GO) and Kyoto Encyclopedia of Genes and Genomes (KEGG) pathways enrichment analyses were performed using the Metascape database (https://metascape.org/). The top 20 predicted KEGG pathways, related metabolites, and targets of YCSND for the treatment of liver injury were screened to construct a metabolites-target-pathway (M-T-P) network using Cytoscape 3.8.2. Furthermore, we screened five typical KEGG pathways to construct a target-pathway (T-P) network using an online website (https://www.bioinformatics.com.cn).

### 2.11 Integrated metabonomics and network analysis

Targets of the metabolites *in vivo* affected by YCSND were predicted using the SWISS database (http://www.swisstargetprediction.ch/). Overlapping targets were obtained among those from the metabolites and network prediction. These targets played a key role in YCSND against ALI.

### 2.12 Data analysis

Each experiment was performed independently at least three times, and data are expressed as mean ± standard deviation (SD). Statistical analysis was performed using GraphPad Prism (version 8.0) and SPSS 22.0. Statistical significance of differences was assessed with Student’s *t*-test for two groups or one-way ANOVA for multiple groups. Differences were considered significant when *p* < 0.05.

## 3 Results

### 3.1 Compositions and antioxidant activity of YCSND

**FIGURE 2:**
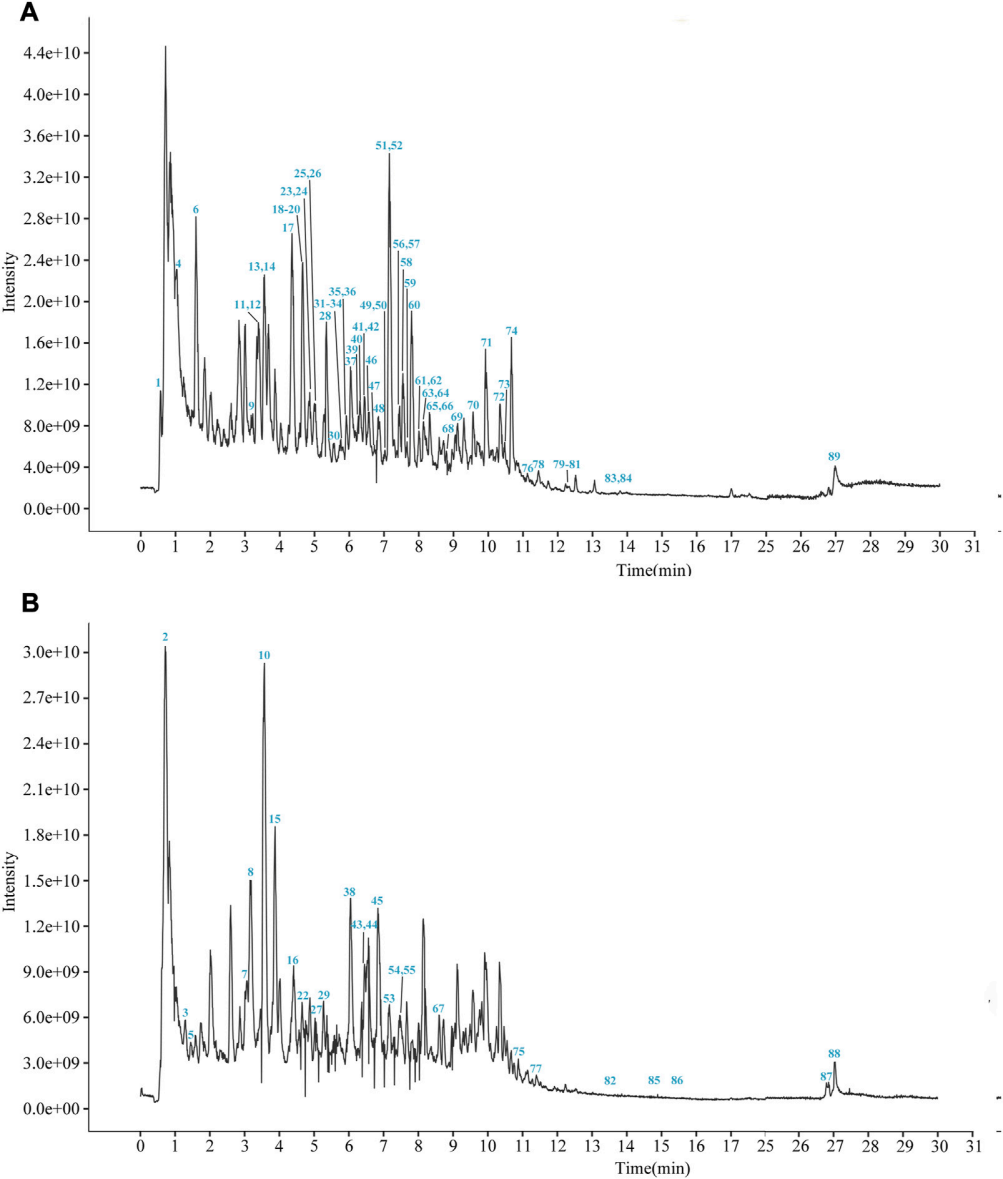
The total ion current chromatogram of YCSND in positive mode **(A)** and negative mode **(B)**.

YCSND contained polysaccharides (408.55 ± 15.04 mg Glu/g), flavonoids (92.70 ± 3.37 mg RE/g), and polyphenols (39.76 ± 0.83 mg GAE/g) (Supplementary Table S3). Additionally, YCSND showed excellent antioxidant activity against several free radicals, including DPPH, ABTS, O^2−^ and ·OH (Supplementary Table S3), which was partly attributed to its polysaccharide, flavonoid, and polyphenol contents (Zhang Q. et al., 2022; Ziyatdinova and Kalmykova, 2023). Moreover, UHPLC-QE-MS identified 89 metabolites, including 20 flavonoids, 18 terpenoids, 14 phenols, 11 phenylpropanoids, 11 alkaloids, three chalcones, and four coumarin derivatives (Supplementary Table S4; Figure 2). Furthermore, key metabolites in YCSND were identified and quantified using HPLC. Supplementary Figure S2 shows the chromatograms of the primarily identified and quantified metabolites of YCSND, and Table 1 lists their specific contents. The main metabolites of YCSND include chlorogenic acid, scoparone, 6-gingerol, 6-shogaol, liquiritin and glycyrrhizic acid. The content of diester alkaloids (neoaconitine, hypaconitine, and aconine) in YCSND is 0.008 mg/g, which is significantly below the pharmacopeia standard of 0.01%. This indicates that YCSND has minimal toxicity. Notably, the metabolites of YCSND included glycyrrhizic acid, chlorogenic acid, 6-gingerol and scoparone, which have been reported to possess antioxidant activity (Merzouk et al., 2017; Huo et al., 2020; Hsueh et al., 2021; Liu et al., 2023). Overall, it could be speculated that these abundant metabolites may contribute to the antioxidant activity of YCSND.

### 3.2 Effects of YCSND on CCl_4_-induced HepG2 cell damage

**FIGURE 3:**
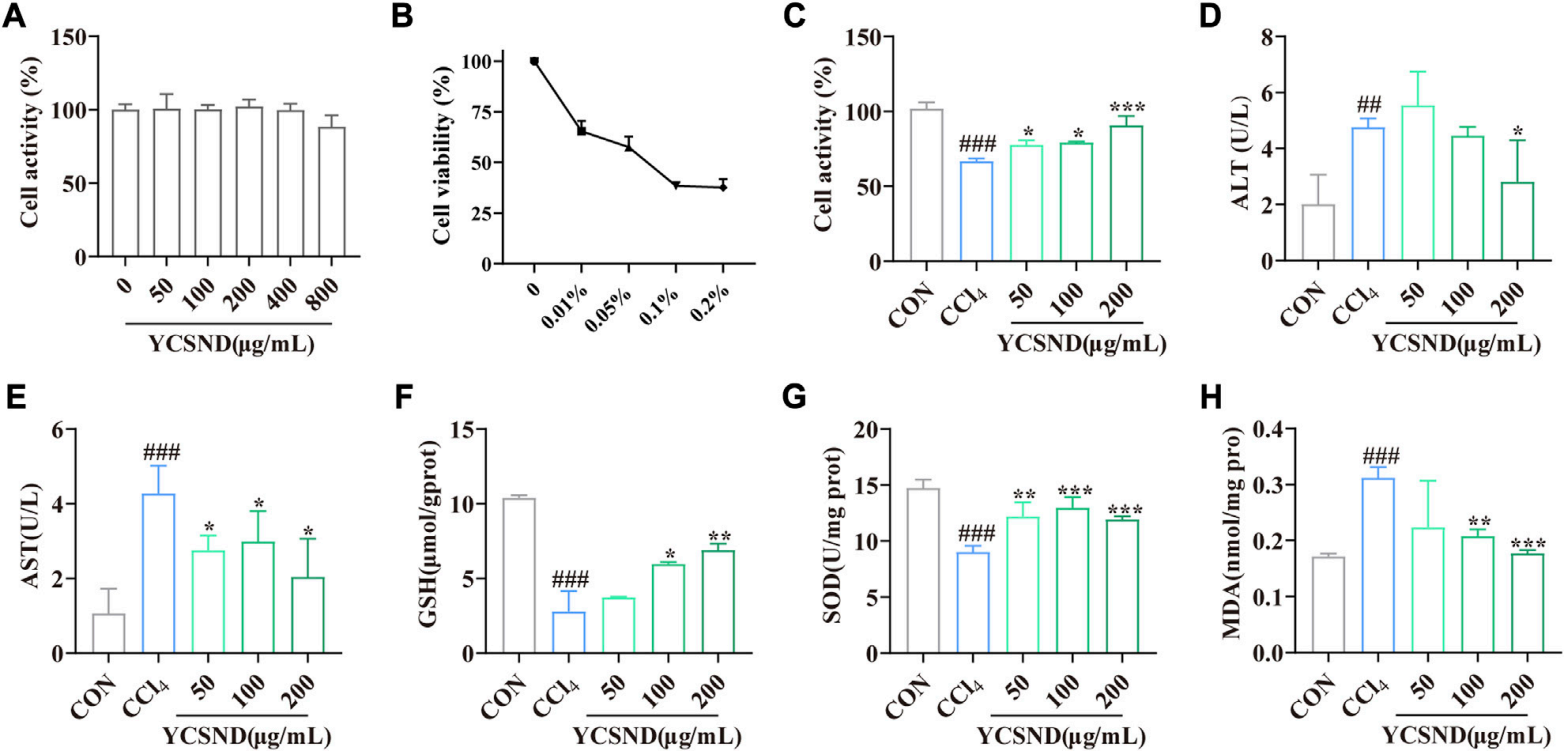
Effect of Yinchen sini decoction (YCSND) on CCl_4_-treated HepG2 cells. **(A)** HepG2 cell viability after YCSND treatment. **(B)** Effect of CCl_4_ treatment at different concentrations on HepG2 cell viability. **(C)** HepG2 cell viability following treatment with YCSND and 0.05% CCl_4_. **(D–H)** Effect of YCSND pretreatment on ALT, AST, GSH, SOD, and MDA contents in CCl_4_-treated HepG2 cells. ^#^ *p <* 0.05, ^##^ *p <* 0.01, ^###^ *p <* 0.001 vs. CON group; ^*^ *p <* 0.05, ^**^ *p <* 0.01, ^***^ *p <* 0.001 vs. CCl_4_ group.

YCSND at concentrations of 0–800 μg/mL had no significant (*p* > 0.05) cytotoxic effect on HepG2 cells (Figure 3A); therefore, subsequent experiments were performed using 50, 100, and 200 μg/mL of YCSND. Additionally, an appropriate CCl_4_ concentration of 0.05% was selected to induce cell damage, which reduced cell viability to approximately 50% (Figure 3B). Expectedly, pretreatment with 50, 100, and 200 μg/mL of YCSND reversed CCl_4_-induced cell damage by significantly improving cell viability by 10.87, 12.84, and 24.10%, respectively, compared with the CCl_4_ group (Figure 3C). Additionally, we examined the protective effects of YCSND against CCl_4_-induced cell damage using biochemical indices. CCl_4_-treated cells had significantly higher extracellular AST and AST, and MDA contents but lower intracellular GSH and SOD levels (Figures 3D–H). However, pretreatment with 50, 100, and 200 μg/mL of YCSND reversed the changes in these indices, especially at a dose of 200 μg/mL (Figures 3D–H), indicating that YCSND could reduce CCl_4_-induced HepG2 cell damage.

### 3.3 Effects of YCSND on ALI in mice *in vivo*

**FIGURE 4:**
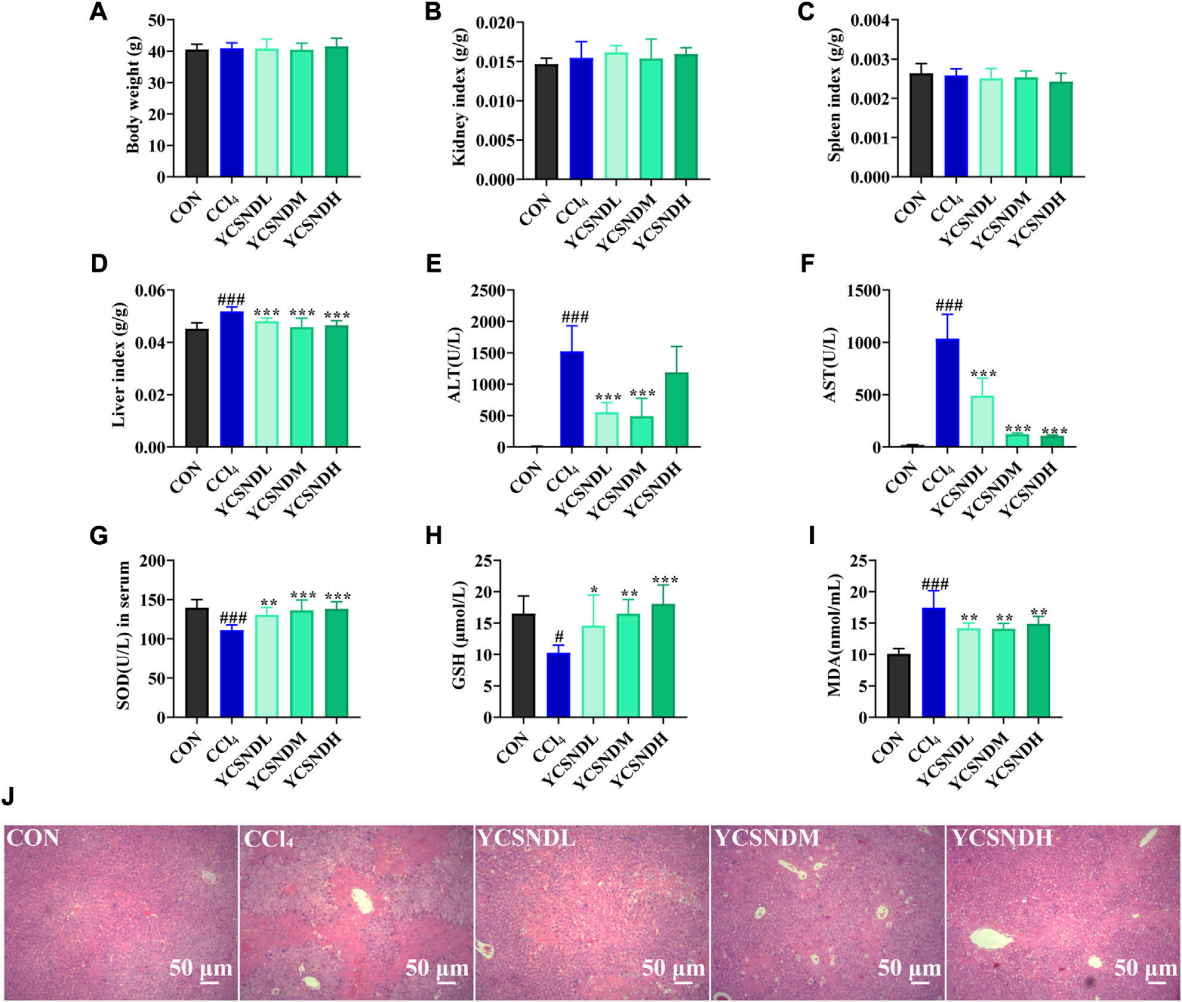
Effects of Yinchen sini decoction (YCSND) on CCl_4_-induced acute liver injury (ALI) mice. **(A-D)** The body weight, kidney index, spleen index and liver index. **(E-I)** The serum of ALT, AST, SOD and GSH levels, and MDA contents. **(J)** H&E staining of liver tissue sections. *#p* < 0.05, *##p* < 0.01, *###p* < 0.001 vs. CON group; *p < 0.05, **p < 0.01, ***p < 0.001 vs. CCl_4_ group.

To study the effects of YCSND on ALI *in vivo*, a mouse model of CCl_4_-induced ALI was generated. Physiological assessment showed that there were no significant differences in body weight, spleen index and kidney index among the groups (Figures 4A–C). However, liver index was significantly higher in the CCl_4_ group than in the CON group, which was significantly reduced by YCSND treatment at all doses (Figure 4D). Changes in serum ALT and AST activities are vital indices for evaluating the degree of ALI. ALT and AST levels were significantly higher in the CCl_4_ group, indicating that the ALI model was successfully established (Figures 4E, F). Compared with the model group, ALT and AST levels were significantly lower in all YCSND treated groups, except for the YCSNDH group, which exhibited a decreasing trend in ALT but not significant (Figures 4E, F). Additionally, YCSND suppressed CCl_4_-induced oxidative stress by reducing MDA content and increasing serum GSH and SOD levels compared with the CCl_4_ group (Figures 4G–I). Overall, these results confirmed that YCSND protects against CCl_4_-induced ALI by regulating biochemical indices.

### 3.4 Effects of YCSND on hepatic histopathology

Compared with serum enzyme activities, hepatic histopathology could be more intuitive in reflecting the degree of liver damage. In the CON group, a normal hepatocytic cell structure was observed with the following representative characteristics: uniform arrangement of hepatocytes, rich hepatocytic cytoplasm, clear cellular nucleoli, and prominent central veins (Figure 4J). In contrast, the CCl_4_ group showed severe pathological changes, including nuclear pyknosis, extensive necrosis, and inflammatory infiltration around the central vein (Figure 4J). However, YCSND treatments ameliorated the histopathological changes associated with CCl_4_-induced liver injury to some extent, especially the YCSNDM and YCSNDH groups, which showed reduced inflammatory infiltration and necrosis compared with that in the CCl_4_ group (Figure 4J).

### 3.5 ^1^H-NMR metabolomics analysis

**FIGURE 5:**
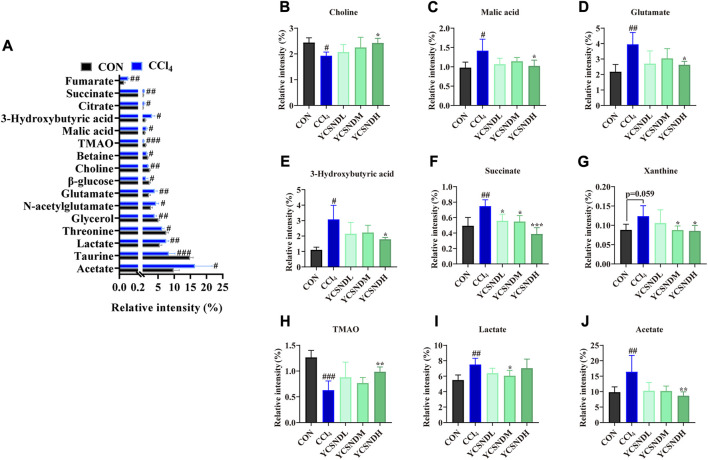
Differential metabolites between the CCl_4_ group and CON group or YCSND group, based on ^1^H-NMR. **(A)** The trend of differential metabolites between CCl_4_ and CON groups. **(B–J)** The relative contents of choline, malic acid, glutamate, 3-hydroxybutyric acid, succinate, xanthine, TMAO, lactate, and acetate, based on ^1^H-NMR analysis. #*p* < 0.05, ##*p* < 0.01, ###*p* < 0.001 vs. CON group; *p < 0.05, **p < 0.01, ***p < 0.001 vs. CCl_4_ group.

^1^H-NMR metabolomic was used to distinguish differential metabolites in the liver of the mice. OPLS-DA score plots demonstrated a clear separation between the CCl_4_ group and the other groups, indicating an abnormal metabolic profile caused by CCl_4_ (Supplementary Figures S3A–D). To prevent overfitting of the model between the CON and CCl_4_ group, a 200-times permutation test was employed. The Q2 values was <0.05, indicating that the OPLS-DA model (R^2^X = 0.854, R^2^Y = 0.998, Q^2^ = 0.898) was reliable (Supplementary Figures S3E). S-plot and VIP values were applied to screen differential metabolites based on VIP >1 and *p* < 0.05 (Supplementary Figures S3F), a total of 16 differential metabolites were altered by CCl_4_ treatment vs. CON groups, among which nine were upregulated and seven were downregulated (Figure 5A). Interestingly, pretreatment with YCSND significantly affected nine metabolites, including choline, malic acid, glutamate, 3-hydroxybutyric acid (3HB), succinate, trimethylamine oxide (TMAO), acetate, lactate, and xanthine (Figures 5B–J), indicating that YCSND improved the CCl_4_-induced abnormal hepatic metabolism. High lactate levels are indicative of CCl_4_-induced liver toxicity (Zira et al., 2013). Our results showed that a medium dose of YCSND significantly reduced hepatic lactate content. Li et al. reported elevated acetate and 3HB levels in a CCl_4_-induced liver injury model (Li et al., 2017), which is consistent with the results of the present study. Choline is a key component of the cell membrane. In our study, choline content was significantly lower in the CCl_4_ group, which is consistent with previous findings (Li et al., 2014; Sun et al., 2020). Patients with cirrhosis had decreased levels of TMAO and choline deficiency could contribute to reduce TMAO levels in liver cirrhosis (van den Berg et al., 2022). TMAO content was significantly lower in the CCl_4_ group compared with CON group. These data show that YCSND alleviated ALI by improving hepatic endogenous metabolite profile.

### 3.6 Network analysis

#### 3.6.1 Target prediction of YCSND metabolites against ALI

A total of 763 targets of 89 metabolites from the SWISS database and 1,432 targets of liver injury from public databases were identified after removing duplicate targets (Supplementary Material S1). Venn diagram showed 229 overlapping targets, which were identified as key targets of YCSND in liver injury (Supplementary Figure S4A). A network of “metabolite-overlapped target” relationship was constructed and shown in Supplementary Figure S4B.

#### 3.6.2 KEGG pathway and GO enrichment

**FIGURE 6:**
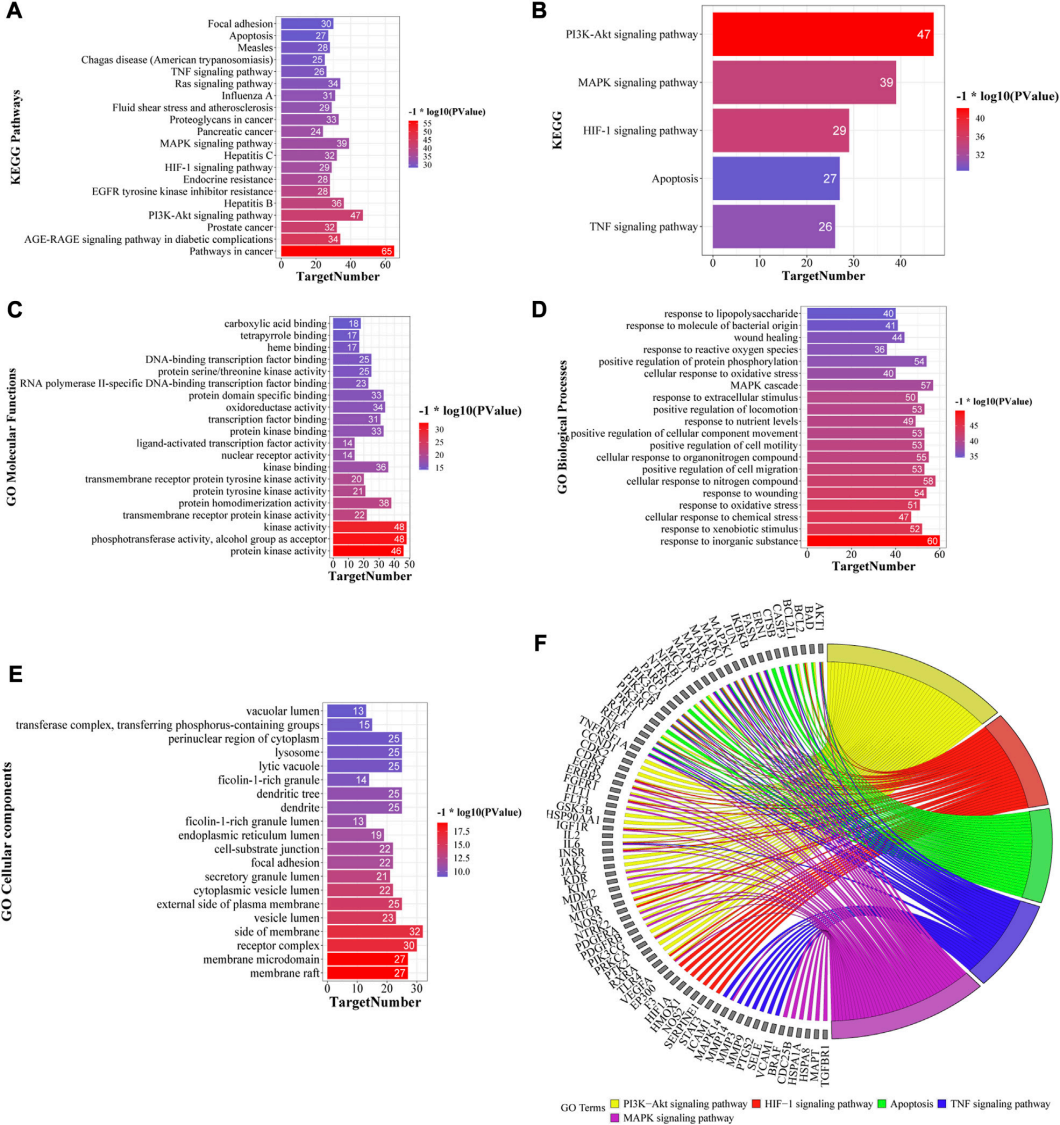
Top 20 KEGG pathways **(A)** and five key KEGG pathways **(B)** of Yinchen sini decoction (YCSND) in liver injury. **(C–E)** Go enrichments including the top 20 of cellular component (CC), molecular Function (MF) and biological process, (BP). **(F)** Target-pathway network of five key KEGG pathways, including PI3K-AKT and HIF-1 signaling pathways, apoptosis, and TNF and MAPK signaling pathways.

GO and KEGG pathway enrichment analyses were performed using the Metascape database (Supplementary Material S2, S3), and the top 20 KEGG pathways based on *p*-value are shown in Figure 6A. KEGG pathway analysis showed that several disease-related pathways were enriched, such as pathways in cancer, hepatitis B and C, and influenza A. We focused on five key KEGG pathways (Figure 6B) that were closely related to the mechanism of liver-injury, according to the literature (Zhou et al., 2020; Abdelhamid et al., 2021). Overall, these results suggest that YCSND may play a key role in ALI prevention by regulating several pathways. GO enrichment analysis showed that the targets were mainly enriched in the functions of cell membrane and organelles, including the side of the membrane, receptor complex, and membrane microdomain, in the “cellular components (CC)” category; in kinase activities and cell factor binding, including kinase, protein kinase, and transcription factor binding, in the “molecular functions (MF)” category; and the physiological state of cells and immune responses in the “biological processes (BP)” category (Figures 6C–E).

#### 3.6.3 Construction of metabolite-target-pathway (M-T-P) network

A M-T-P network was constructed to elucidate the relationships among the top 20 KEGG pathways, related targets and metabolites. The network contained 1,451 edges and 208 nodes, including 78 metabolites, 110 targets, and 20 KEGG pathways (Supplementary Figure S5A). Additionally, the target-pathway (T-P) and M-T-P networks were constructed using five key pathways related to ALI (Figure 6F; Supplementary Figure S5B). The T-P network showed that the targets with high degrees in these five pathways were AKT1, NfκB1, MAPK3, PI3KCA, PI3KCB, PI3KR1 and RELA, among which AKT1, PI3KCA, PI3KCB and PI3KR1 are key genes involved in the PI3K-AKT signaling pathway. RELA and NfκB1 are crucial targets involved in the NFκB pathway which regulates inflammatory cytokines. Therefore, we speculated that the PI3K-AKT signaling pathway and inflammation regulation may be involved in the effects of YCSND against ALI. The M-T-P network showed that the five metabolites with the highest degree values were 6-gingerol (degree, 46), scoparone (degree, 41), galangin (degree, 40), 8-shogaol (degree, 40), and morusin (degree, 39). Notably, HPLC confirmed that YCSND has a high scoparone content. Scoparone is the active ingredient of Artemisia capillaris Thunb. [Asteraceae; Artemisiae scopariae herba] and has positive therapeutic effects on liver diseases (Hui et al., 2020). Overall, the results of the network analysis indicated that YCSND had multifaceted synergistic effects against ALI by integrating multiple metabolites, targets, and multi-pathways against ALI, which is consistent with the theory of TCM.

### 3.7 The molecular mechanism of YCSND against acute liver injury (ALI)

**FIGURE 7:**
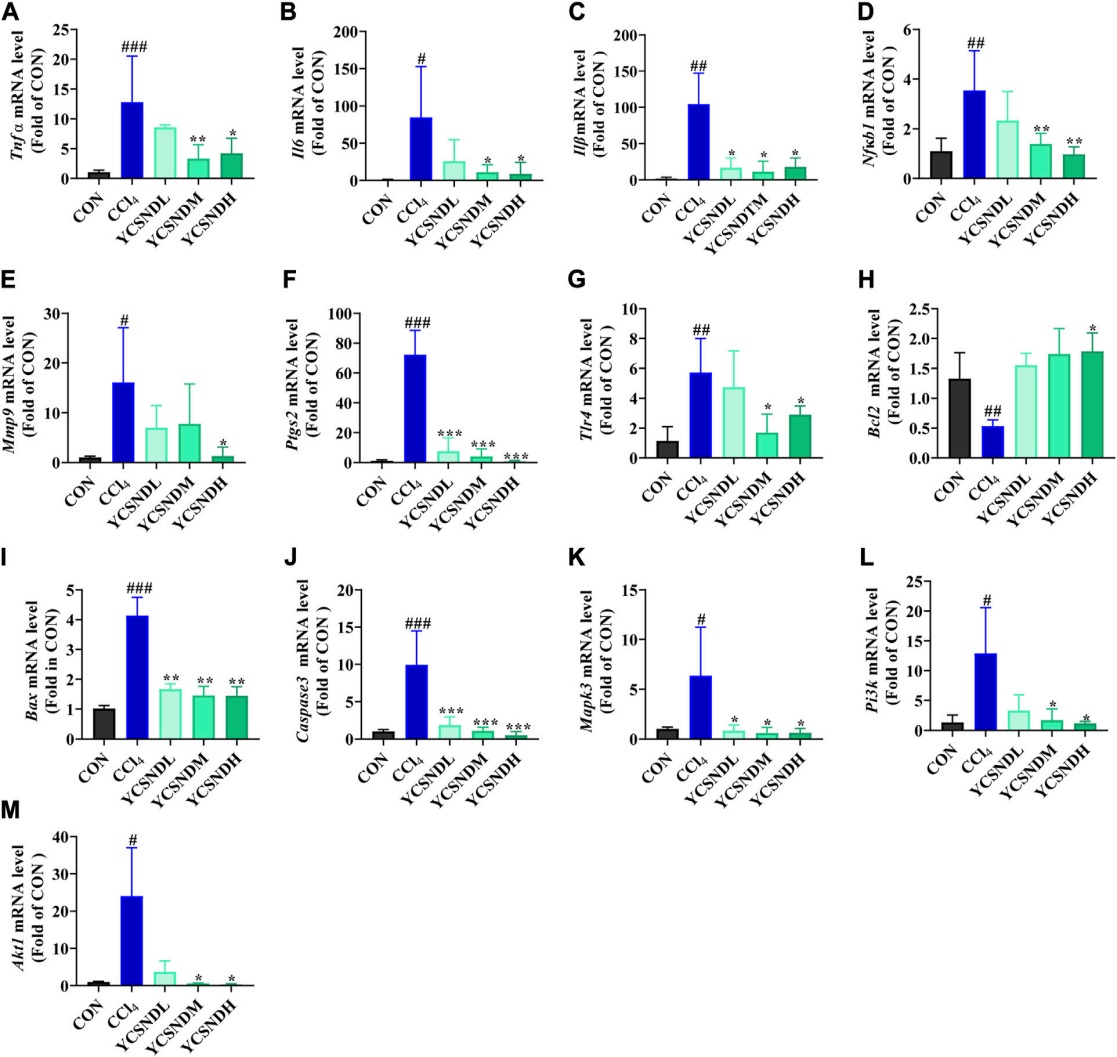
The results of experimental verification *in vivo*. **(A-M)** mRNA expression levels of *Tnf α*, *Il6*, *Il1β*, *Nfκb1*, *Bax*, *Caspase3*, *Bcl2*, *Tlr4*, *Mmp9*, *Ptgs2*, *Mapk3*, *Pi3k*, and *Akt1*. *#p* < 0.05, *##p* < 0.01, *###p* < 0.001 vs. CON group; *p < 0.05, **p < 0.01, ***p < 0.001 vs. CCl_4_ group.

To explore the molecular mechanisms of YCSND in ALI, we identified multiple targets from five ALI-related pathways predicted by network analysis. These targets included *Pi3k*, *Akt1*, *Mapk3*, *Tlr4*, *Il6*, *Tnf*, *Nfκb1*, *Bcl2*, *Caspase3*, *Mmp9*, *Ptgs2, Stat3*, *Vegfa,* and *Egfr*. Additionally, the important inflammatory cytokines *Il1β* and apoptotic gene *Bax* were also included. Network predictions showed that YCSND may have anti-inflammatory activity, which was consistent with the results on the *in vivo* experiments. YCSND demonstrated excellent anti-inflammatory effects against ALI by reducing the mRNA levels of the pro-inflammatory factors *Il6*, *Il1β*, *Tnfα* and the upstream inflammatory regulator *Nfκb1* (Figures 7A–D). TLR4, PTGS2, and MMP9 are closely associated with inflammatory responses, and TLR4 regulates NFκB activation, which can promote PTGS2 expression. Inflammatory response and tissue damage caused by CCl_4_ in mice could induce the overexpression of MMP9 (Hafez et al., 2017). CCl_4_ increased *Tlr4*, *Ptgs2* and *Mmp9* mRNA levels in the liver (Figures 7E–G), which was consistent with previous findings (Leung et al., 2011; Zhao D. et al., 2022). However, YCSND pretreatment reversed CCl_4_-induced changes in inflammation-related genes, but did not significantly affect *Vegfa*, *Efgr*, and *Stat3* mRNA levels, which are mainly involved in the HIF-1 signaling pathway (Supplementary Figures S6A–C). Furthermore, we examined changes in the expression levels of the apoptosis-related genes *Bcl2*, *Caspase3*, and *Bax*. CCl_4_ upregulated the expression of the pro-apoptotic genes *Bax* and *Caspase3* and downregulated the anti-apoptotic gene *Bcl2* (Figures 7H–J). In contrast, YCSND pretreatment reversed CCl_4_-induced changes in the expression of apoptosis-related genes, indicating an anti-apoptotic effect against ALI. The activation of mitogen-activated protein kinase (MAPK) can accelerate apoptosis by inhibiting *Bcl2* expression and inducing the activation of caspase family proteins (Dong et al., 2015). *Mapk3* expression was upregulated in the CCl_4_ group, which was reversed by YCSND treatment (Figure 7K). Additionally, the PI3K-AKT signaling pathway was the most significant pathway closely associated with ALI in computational prediction. Compared with the CCl_4_ group, YCSND treatment inhibited *Pi3k* and *Akt* mRNA expression (Figures 7L, M). These results suggest that YCSND improved ALI by regulating PI3K-AKT signaling pathway and ameliorating inflammation and apoptosis.

### 3.8 Integration of network analysis and metabolomics

**FIGURE 8:**
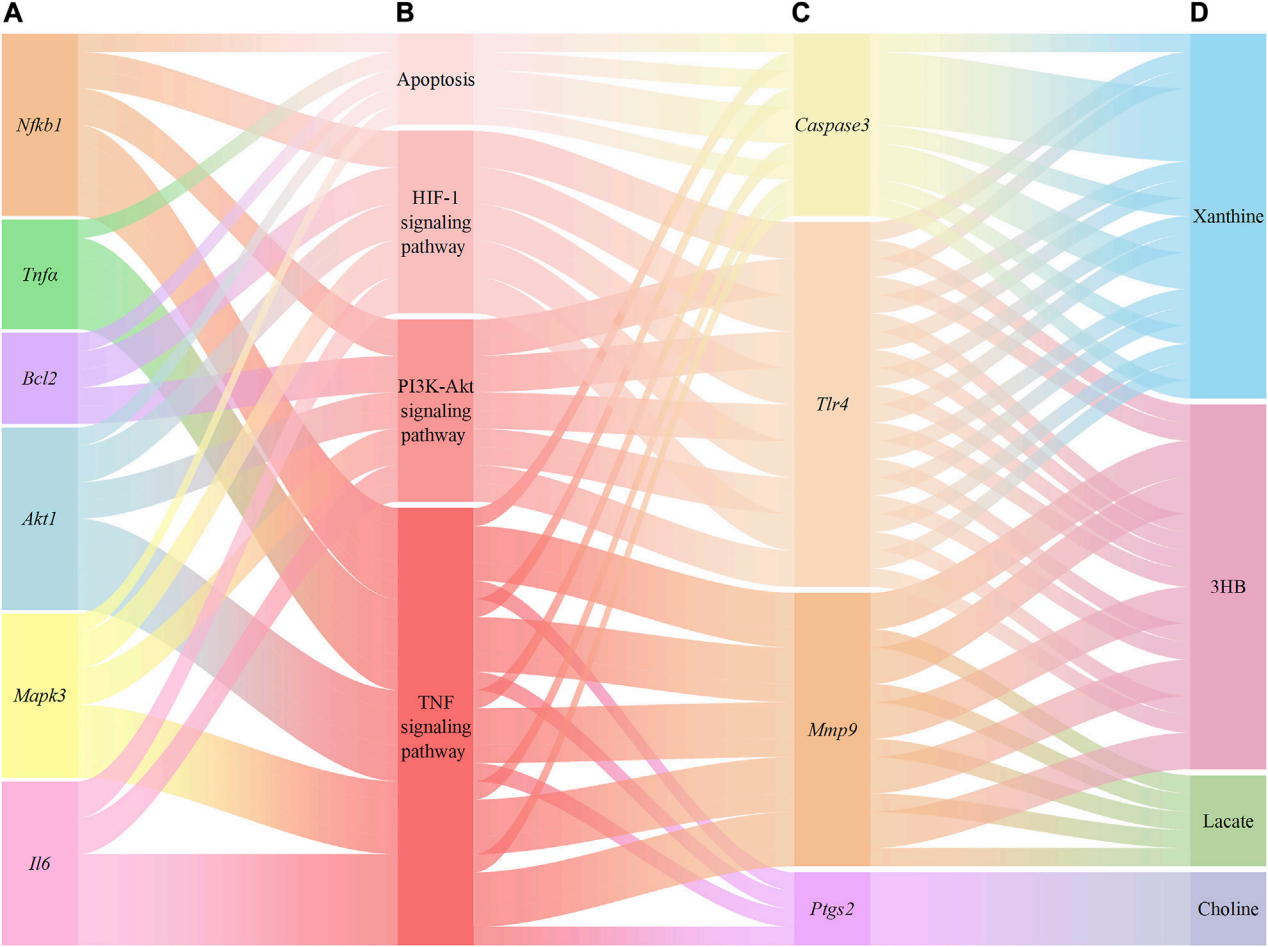
The relationship between the targets of network pharmacological prediction and metabolites regulated by Yinchen Sini Decoction (YCSND). **(A)**, the validated targets from pharmacological prediction; **(B)**, pathways associated with acute liver injury (ALI); **(C)**, the validated crossover targets; **(D)**, metabolites regulated by YCSND.

A total of 267 targets from 8 metabolites were altered by YCSND, except for acetate (no target was found), based on the SWISS database. Additionally, we identified 19 overlapping targets from the metabolites altered by YCSND and five pharmacological prediction pathways related to ALI (Supplementary Material S4). We also verified that YCSND regulated the targets of *Caspase3*, *Tlr4*, *Mmp9*, and *Ptgs2*, which were mainly linked to the metabolites including xanthine, 3HB, lactate, and choline. *Tlr4*, *Mmp9*, and *Ptgs2* are related to inflammatory response and *Caspase3* is a typical target associated with apoptosis. Finally, we constructed a network to link the metabolites changed by YCSND *in vivo*, crossed targets (metabolites and network analysis), pathways related to ALI, and targets related to pathways affected by YCSND (Figure 8).

## 4 Discussion

Over the years, several clinical trials have demonstrated the hepatoprotective potential of YCSND in hepatobiliary diseases (Wang et al., 2008); however, the underlying molecular mechanisms remain unclear. The theory of TCM emphasizes the holistic and synergistic effects of multiple metabolites of YCSND. The main metabolites of YCSND, chlorogenic acid, 6-gingerol and scoparone, have been reported to possess therapeutic effects against ALI (Merzouk et al., 2017; Hui et al., 2020; Liu et al., 2023). In the present study, we elucidated the mechanism of the hepatoprotective effects of YCSND using integrating network analysis and metabolomics.

The metabolites of TCM formulas are not simple combinations of plant-derived metabolites; instead, they are influenced by the source of botanical drugs and processing techniques (Li Z. et al., 2022). There was a noticeable discrepancy between the predicted outcomes based on the actual compositions of TCM and the database (Li Z. et al., 2022). Although the predicted YCSND metabolites based on the database were not included in the present study, there were notable discrepancies between the network analysis results obtained from the predicted components and the actual metabolites of YCSND. We attempted to predict the metabolites of YCSND based on a previous database (TCMSP database) and identified 105 effective metabolites, UHPLC-QE-MS identified only 89 metabolites of YCSND detected by UHPLC-QE-MS, indicating that only a small fraction of the actual metabolites of YCSND were consistent with the predictions. Additionally, the top 20 KEGG pathways based on the predicted metabolites appeared unreliable, as these pathways were mainly related to cancer and diabetes, rather than the five pathways related to ALI based on predictions using the actual metabolites. Overall, predictions based on the actual metabolites of YCSND could minimize the inherent prediction bias in network analysis.

Biomarkers are effective tools for monitoring disease progression and assessing treatment effectiveness (Borém et al., 2023). CCl_4_-induced ALI can lead to abnormal accumulation or depletion of hepatic endogenous metabolites in the body, affecting a range of metabolic pathways, such as amino acid metabolism, energy metabolism, and lipid metabolism (Zira et al., 2013; Li et al., 2017). Choline deficiency may be related to CCl_4_-induced breakdown of cell membrane structure, resulting in the outflow of ALT from the liver into the serum (Li et al., 2014). The tricarboxylic acid (TCA) cycle, an important energy metabolism pathway, is the hub for amino acid and glycolipid metabolism. CCl_4_ can induce abnormal energy metabolism by damaging the hepatocellular mitochondria and inhibiting enzyme activity during the TCA cycle (Zira et al., 2013). However, YCSND can restore malic acid and succinate, which are intermediate metabolites of the TCA cycle. Moreover, glutamate can participate in the glutamic acid and glutamine cycles, and glutamine is degraded into glutamate and ammonia by glutaminase. Liver injury can lead to impaired ammonia metabolism in the body, potentially resulting in hyperammonemia (Jimenez-Torres et al., 2021); however, a decrease in glutamate can reverse this condition. Moreover, low levels of glutamate are beneficial for ROS clearance and the improvement of antioxidant capacity (Demircan et al., 2014; Chen et al., 2023). The excessive oxidation of fatty acids can increase the end-product acetate and induce the accumulation of ketone bodies in the CCl_4_-induced ALI model (Huo et al., 2016), which are consistent with our results of increasing acetic acid and ketone body 3HB in CCl_4_ group. Notably, we showed that pretreatment with YCSND reversed these changes. Overall, these results support the hypothesis that YCSND has a positive effect on abnormal metabolism in the liver of mice.

The relationship between genes and metabolites is bidirectional and complex, with genes affecting metabolites, and *vice versa*. In the present study, we integrated network analysis and metabolomics to link the differential metabolites to gene targets. Figure 8 depicts the potential associations between metabolites and target genes. Xanthine oxidase converts hypoxanthine to xanthine, which eventually forming uric acid. Elevated xanthine concentrations can contribute to the formation of uric acid, posing potential health risks; the increase of xanthine can activate the expression of CASPASE3, whereas xanthine oxidase inhibitors had the opposite effect (Genesca et al., 2002; Park et al., 2010). High levels of 3HB could initiate ketosis, resulting in the activation of TLR4/NFκB to induce high expression of inflammation under a state of ketosis (Li et al., 2017). Moreover, the inhibition of TLR4 may be beneficial for the regulation of ketone body levels and the decrease in 3HB levels. Choline has been reported to treat LPS-induced inflammation in rats by reducing PTGS2 levels (Baris et al., 2021). And long-term choline deficiency diet can lead to increased hepatic PTGS2 levels (Motino et al., 2016; He et al., 2022). In this study, choline levels increased and *Ptgs2* mRNA level decreased after treatment with YCSND, which is consistent with previous reports (Motino et al., 2016; He et al., 2022). Pyruvate generates lactic acid under the action of lactate dehydrogenase, and lactate dehydrogenase knockout reduces the level of MMP9 (Wang et al., 2017). Moreover, a positive correlation has been reported between lactic acid level and MMP9 (Park et al., 2005; Duda et al., 2020). Overall, these data support the potential association between targets and metabolites that could be regulated by YCSND.

The metabolism-related targets identified in the present study have been reported to be involved in inflammation and apoptosis, which are important regulatory targets in ALI. Exposure to CCl_4_ can activate Kupffer cells in the liver, which can promote the expression of pro-inflammatory factors through TLR4/NFκB signaling (Hsieh et al., 2021; Sun et al., 2022). Additionally, the TNF signaling pathway, which is related to the inflammatory response is activated during CCl_4_-induced ALI (Zhang J. X. et al., 2022). In the present study, YCSND reduced hepatic inflammation by inhibiting the mRNA expression of the pro-inflammatory factor *Il6*, *Il1β*, and *Tnfα* and the inflammatory regulators *Nfκb1* and *Tlr4*. These results suggest that YCSND effectively inhibited the TNF signaling pathway by downregulating the typical target genes of the pathway, including *Il6*, *Tnfα*, and *Nfkb1*. The upregulation of the pleiotropic cytokine TNFα not only promotes inflammation but also accelerates cell apoptosis (Dong et al., 2016). Additionally, excessive free radicals produced during the hepatic metabolism of CCl_4_ can trigger mitochondrial dysfunction and induce hepatic apoptosis. An increase in the pro-apoptotic protein BAX can enhance mitochondrial permeability and activate CASPASE3 to induce apoptosis (Wang Y. et al., 2023). In the present study, there was a decrease in the expression of the anti-apoptotic *Bcl2* mRNA level in damaged cells, which was reversed by treatment with YCSND. Overall, these results indicate that YCSND reduced inflammation and inhibited hepatocyte apoptosis.

Research evidence has shown that the PI3K-AKT pathway contributes to the development of liver injury and fibrosis, including the activation of hepatic stellate cells, synthesis and degradation of the extracellular matrix, and regulation of matrix metalloproteinase (MMP) activity (Gong et al., 2020). TCM has been shown to possess therapeutic effects in ALI by inhibiting the PI3K-AKT pathway (Zhao et al., 2018; Gong et al., 2020; Wang J. M. et al., 2023). For instance, Qinggan Huoxue Recipe attenuated ethanol-induced ALI by downregulating *Pi3k* and *Akt1* mRNA levels. Additionally, the Dahuang Zhechong pill ameliorated CCl_4_-induced liver fibrosis by reducing the protein levels of PI3K and phosphorylated AKT and inhibiting the proliferation of HSC, and exerting a synergistic effect with the PI3K inhibitor LY294002. Zhao et al. reported a relationship between the PI3K/AKT/Raptor/Rictor signaling pathway and apoptosis and observed that Erzhi Pill can ameliorate ALI by inhibiting the pathway to reduce hepatic apoptosis (Zhao et al., 2018). Moreover, AKT phosphorylation can exacerbate ALI, whereas AKT inhibitors can suppress hepatic apoptosis by inhibiting the PI3K/AKT/FXR axis *in vitro* (Jiang et al., 2023). The PI3K-AKT signaling pathway is also vital in the regulation of inflammation, especially the PI3K/AKT/NF-κB axis (Zhu et al., 2017; Yang et al., 2020). Phosphorylated AKT enhances the nuclear transcription of NFκB and the release of pro-inflammatory cytokines, and these effects are blocked by PI3K inhibitors, thereby inhibiting hepatocyte apoptosis, reducing inflammation, and alleviating liver damage (Gong et al., 2020). Glycyrrhizic acid (Niu et al., 2022), scoparone (Liu et al., 2020), isorhamnetin (Li et al., 2015), quercetin (Yang et al., 2020), and luteolin (Jiang et al., 2023) have been reported to regulate the PI3K/AKT axis to improve ALI. And these metabolites were detected in YCSND in our study. In the present study, YCSND inhibited *Pi3k* and *Akt1* mRNA expression, indicating that YCSND can regulate the PI3K-AKT pathway to inhibit hepatic apoptosis and inflammatory responses in ALI.

This study had some limitations. Although the accuracy of the network prediction has improved, there was still heterogeneity in the experimental validation and prediction results in the present study. YCSND had only minimal effects on some of the predicted targets, such as *Vegfa*, *Egfr*, and *Stat3*. We suggested that network analysis should be based on the actual constituents of the TCM, rather than botanical drug information from existing databases. However, network analysis still relies heavily on databases, and this inherent bias caused by the databases is a common issue in network analysis. The completeness and timeliness of the Swiss database can particularly impact our study results. For example, delayed data updates and the abundance of potential targets for well-known active metabolites like quercetin with the less targets of new metabolites can lead to result in false positives or false negatives. To narrow down these biases, continuous improvement and timely information updates of databases should be the common goal of future network analysis research. Moreover, network analysis predictions may not comprehensively reflect the intricate interactions and mechanisms of drugs within the body. Therefore, experimental verification is necessary to ensure the authenticity of network predictions. Meanwhile, regarding the connection between network analysis and metabolism, the target genes related to changes in differential metabolites require further investigation. We validated the highly significant PI3K signaling pathway predicted by network analysis through RT-qPCR analysis. In future studies, other signaling pathways predicted by network analysis should be further considered to expand the molecular mechanisms of YCSND in ALI.

## 5 Conclusion

**FIGURE 9:**
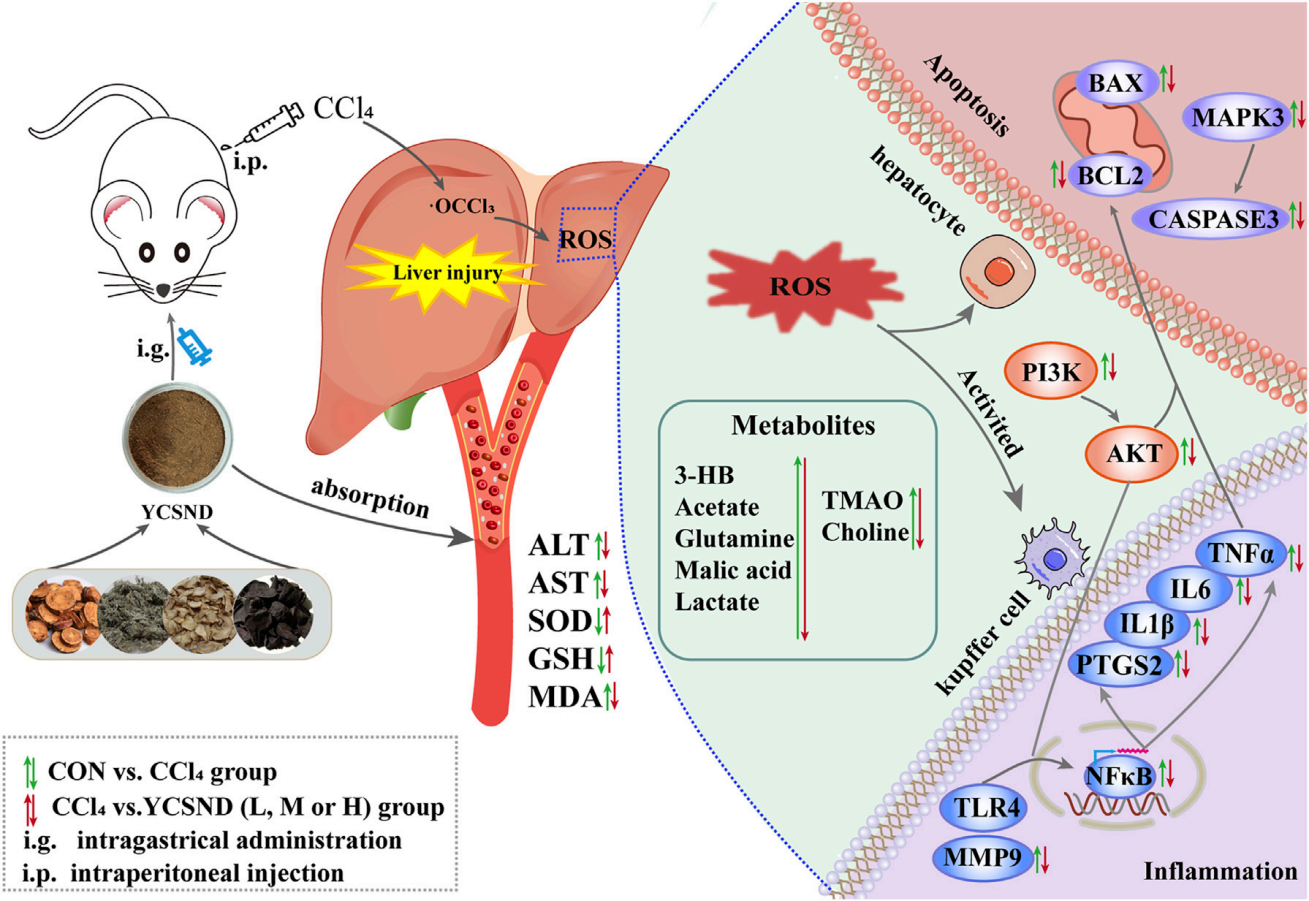
The molecular mechanism of Yinchen sini decoction (YCSND) against acute liver injury (ALI).

In the present study, integrated network analysis and ^1^H-NMR metabolomics revealed key targets (*Tlr4*, *Ptgs2*, *Mmp9,* and *Caspase3*) and related metabolites (choline, xanthine, lactate, and 3HB) regulated by YCSND in ALI treatment. Our results indicated that YCSND ameliorated ALI by regulating the PI3K-AKT signaling pathway, reducing inflammatory response, and inhibiting apoptosis (Figure 9). Overall, our findings indicate that YCSND has the potential to alleviate ALI, offering a foundational support for the subsequent development and utilization. We have also pointed out the direction for applying network analysis in TCM formulas, stressing the importance of utilizing real metabolites for network analysis and experimental validation. Enhancing the precision of network analysis presents a challenge that future researchers should collectively address.

## Data Availability

The original contributions presented in the study are included in the article/Supplementary Material, further inquiries can be directed to the corresponding authors.
