# Supplementary material for: Integrated network analysis and metabolomics reveal the molecular mechanism of Yinchen Sini decoction in CCl4-induced acute liver injury
Source: Front Pharmacol. 2023 Sep 25;14:1221046. doi: 10.3389/fphar.2023.1221046 (PMC10561237; doi:10.3389/fphar.2023.1221046)
Supplement: Supplementary file 2 [file DataSheet1.docx]

Supplementary Material

Integrated network analysis and metabolomics reveal the molecular mechanism of Yinchen Sini decoction in CCl_4_-induced acute liver injury

Weiwei Zheng, Chao Shi, Yao Meng, Jian Peng, Yongfei Zhou, Tong Pan, Ke Ning, Qiuhong Xie, Hongyu Xiang

*** Correspondence:** Qiuhong Xie Professor: email: qhxie@jlu.edu.cn;

Hongyu Xiang Professor: email: hyxiang@jlu.edu.cn

# Material methods

## Detection of high-performance liquid chromatography (HPLC) Analysis

### Samples Preparation

The quality of four herbs in YCSND were determined by HPLC and their main components were quantitatively analyzed. According to the 2015 edition of Chinese Pharmacopoeia, the sample preparation of *Artemisia capillaris* Thunb. [Asteraceae; Artemisiae scopariae herba], *Aconitum carmichaelii* Debeaux [Ranunculaceae, Aconiti lateralis radix praeparata], *Zingiber officinale* Roscoe [Zingiberaceae, Zingiberis rhizoma], and honey-fried *Glycyrrhiza glabra* L. [Fabaceae, Glycyrrhizae radix et rhizoma praeparata cum melle] was carried out. [Specifically,](javascript:;) *Artemisia capillaris* Thunb. powder (1 g) was placed into a 50 mL conical flask with a stopper, to which 50 mL of 50% methanol was added, followed by precise weighing. After sonication for 30 min, the weight loss was compensated with 50% methanol. *Zingiber officinale* Roscoe powder (0.25 g) was placed into a 25 mL conical flask with a stopper, to which 50 mL of 75% methanol was added, followed by precise weighing. After sonication for 40 min, the weight loss was compensated with 75% methanol. honey-fried *Glycyrrhiza glabra* L. powder (2 g) was placed into a 100 mL conical flask with a stopper, to which 50 mL of 70% ethanol was added, followed by precise weighing. After sonication for 40 min, the weight loss was compensated with 70% ethanol. *Aconitum carmichaelii* Debeaux powder (2 g) was placed into a 100 mL conical flask with a stopper, to which 3 mL of ammonia water and 50 mL of [isopropanol](javascript:;)-[ethyl acetate](javascript:;) (1/1, v/v) were added, followed by precise weighing. After sonication for 40 min, the weight loss was compensated with [isopropanol](javascript:;)-[dichloromethane](javascript:;) (1/1, v/v). YCSND is prepared according to the methods of its single drug preparation described above, respectively.

### Standard stock solutions preparation

The standard stock solutions of scoparone, [chlorogenic acid](javascript:;), caffeic acid, isochlorogenic acid B, 6-gingerol, 6-shogaol, liquiritin and glycyrrhizic acid were prepared in methanol, and benzoylneaconitine, benzoylaconitine, benzoylhypaconitine, [aconitine](javascript:;), [hypaconitine](javascript:;) and mesaconitine were prepared in [isopropanol](javascript:;)-[dichloromethane](javascript:;) (1/1, v/v). All standards were purchased from DESITE Biotech. (Chengdu, China), with purities exceeding 98%. All stock solutions were stored away from light at 4 °C and were diluted to appropriate [concentration](javascript:;)s using to make the standard curves.

### HPLC conditions

We used four different HPLC procedures to control the quality of single botanical drugs of YCNSD, and establish YCSND fingerprints and determine the contents of its major metabolites. HPLC separation was performed on an Unitary C18 column (4.6 mm × 250 mm, 5 μm) and maintained at 30 °C. Program 1: [acetonitrile](https://www.sciencedirect.com/topics/pharmacology-toxicology-and-pharmaceutical-science/acetonitrile) (solvent A) and 0.05% (v/v) phosphoric acid in distilled water (solvent B) made up the mobile phase. The following gradient was used for elution: 0–30 min, 10%-15% A; 30–45 min, 15%–30% A; 45–60 min, 30%–10% A; 60–65 min, 10% A. The samples were identified at 325 [nm](https://www.sciencedirect.com/topics/pharmacology-toxicology-and-pharmaceutical-science/nitrogen-mustard) (flow rate: 1.0 mL/min). Program 2: [acetonitrile](https://www.sciencedirect.com/topics/pharmacology-toxicology-and-pharmaceutical-science/acetonitrile) (solvent A) and distilled water (solvent B) made up the mobile phase. The following gradient was used for elution: 0–5 min, 35% A; 5–40 min, 35%-70% A; 40–42 min, 70%–80% A; The samples were identified at 240 [nm](https://www.sciencedirect.com/topics/pharmacology-toxicology-and-pharmaceutical-science/nitrogen-mustard) (flow rate: 1.0 mL/min). Program 3：[acetonitrile](https://www.sciencedirect.com/topics/pharmacology-toxicology-and-pharmaceutical-science/acetonitrile) (solvent A) and 0.1% (v/v) phosphoric acid in distilled water (solvent B) made up the mobile phase. The following gradient was used for elution: 0–15 min, 20%-30% B; 15–25 min, 30%–32% B; 25–30 min, 32%–43% A; 30–43 min, 43%-44% B; 43–48 min, 44%-90% B. 48–55 min, 90%-20% B. The samples were identified at 254 [nm](https://www.sciencedirect.com/topics/pharmacology-toxicology-and-pharmaceutical-science/nitrogen-mustard) (flow rate: 1.0 mL/min). Program 4: [acetonitrile](https://www.sciencedirect.com/topics/pharmacology-toxicology-and-pharmaceutical-science/acetonitrile)-[tetrahydrofuran](javascript:;) (25:15, v/v) (solvent A) and 0.1 mol/L [ammonium acetate](javascript:;) in distilled water (solvent B) made up the mobile phase. The following gradient was used for elution: 0–48 min, 15%-26% A; 48–49 min, 26%–35% B; 49–58 min, 35% A; 58–65 min, 35%-15% A. The samples were identified at 235 [nm](https://www.sciencedirect.com/topics/pharmacology-toxicology-and-pharmaceutical-science/nitrogen-mustard) (flow rate: 1.0 mL/min). Program 1 is used to detect scoparone, [chlorogenic acid](javascript:;), caffeic acid and isochlorogenic acid B; program 2 is used to detect 6-gingerol and 6-shogaol; program 3 is used to detect liquiritin and glycyrrhizic acid; and program 4 is used to detect benzoylneaconitine, benzoylaconitine, benzoylhypaconitine, [aconitine](javascript:;), [hypaconitine](javascript:;) and mesaconitine.

## Detection of Compositions of YCSND by UHPLC-QE-MS

In order to clarify the complicated compositions of YCSND, we used a UHPLC-QE-MS to identify active ingredients. In brief, 100 mg of lyophilized powder of YCSND was added to 1 mL pure water. After a 30s vortex, the mixture was followed by ultrasonic in the ice water bath for 5 min. And then the samples were centrifuged at 12000 rpm for 15 min at 4 ℃. Finally, the supernatant was obtained and put in a 2 mL tube for UHPLC-QE-MS analysis. The quality control sample was prepared by mixing an equal aliquot of the supernatants from all of the samples. UHPLC-QE-MS analysis was performed on a Thermos scientific ultra-high performance liquid chromatography vanquish system with a Waters UPLC BEH C18 column (1.7 μm×2.1×100 mm). The flow rate was set at 0.4 mL/min and the sample injection volume was set at 5 μL. The mobile phase consisted of 0.1 % formic acid in water (A) and 0.1 % formic acid in acetonitrile (B). And the multi-step linear elution gradient program was described in followed Supplementary Table S1. The mass spectrometer was operated both in positive and negative ion mode. During each acquisition cycle, the mass range was from 100 to 1500, and the top ten of every cycle were screened and the corresponding data were further analyzed. The flow rate of sheath gas was 45 arbitrary units and the capillary temperature was 400°C. The auxiliary gas was set up to 15 arbitrary units at 400 °C. The capillary voltage was set to 4.0 kV (positive) and -3.8 kV (negative). The raw data processes by XCMS software and the specific steps referred to the published literature (Ai et al, 2021).

**References**

Ai, Q. Wu, M. Battino, W. Bai, and L. Tian, using untargeted metabolomics to profile the changes in roselle (Hibiscus sabdariffa L.) anthocyanins during wine fermentation. Food Chem. 364 (2021) 130425

**Supplementary** **Table S1.** Liquid chromatography mobile phase conditions.

| Time (min) | flow velocity (μL/min) | A% | B% |
| --- | --- | --- | --- |
| 0 | 400 | 95 | 5 |
| 3.5 | 400 | 85 | 15 |
| 6 | 400 | 70 | 30 |
| 6.5 | 400 | 70 | 30 |
| 12 | 400 | 30 | 70 |
| 12.5 | 400 | 30 | 70 |
| 18 | 400 | 0 | 100 |
| 25 | 400 | 0 | 100 |
| 26 | 400 | 95 | 5 |
| 30 | 400 | 95 | 5 |

**Supplementary Table S2.** Sequence of primers used for the RT-qPCR assays.

| **Gene** | **Genes Forward primer (5’-3’)** | **Reverse primer (5’-3’)** |
| --- | --- | --- |
| *Gapdh* | 5’-GTGTTCCTACCCCCAATGTGT-3’ | 5’-ATTGTCATACCAGGAAATGAGCTT-3’ |
| *Tlr4* | 5’-AGCTCCTGACCTTGGTCTTG-3’ | 5’-CGCAGGGGAACTCAATGAGG-3’ |
| *Nfκb1* | 5’CCCTGAGAAAGAAACACAAGG-3’ | 5’-ATGAAGGTGGATGATGGCTAAG-3’ |
| *Tnfα* | 5’-AGACCCTCACACTCAGATCA-3’ | 5’-TCTTTGAGATCCATGCCGTTG-3’ |
| *Il6* | 5’-GTTCTCTGGGAAATCGTGGA-3’ | 5’-TGTACTCCAGGTAGCTA-3’ |
| *Il1β* | 5’-TCCATGAGCTTTGTACAAGGA-3’ | 5’-AGCCCATACTTTAGGAAGACA-3’ |
| *Ptgs2* | 5’-TTCAACACACTCTATCACTGGC-3’ | 5’-AGAAGCGTTTGCGGTACTCAT-3’ |
| *Mmp9* | 5’-CTGGACAGCCAGACACTAAAG-3’ | 5’-CTCGCGGCAAGTCTTCAGAG-3’ |
| *Caspase3* | 5’ATGGAGAACAACAAAACCTCAGT-3’ | 5’-TTGCTCCCATGTATGGTCTTTAC-3’ |
| *Bcl2* | 5’-CTCAGGCTGGAAGGAGAAGAT-3’ | 5’-AAGCTGTCACAGAGGGGCTAC -3’ |
| *Bax* | 5’-GCAAAGTAGAAGAGGGCAACC-3’ | 5’-ACTGGACAGCAATATGGAGCT-3’ |
| *Mapk3* | 5’-TCCGCCATGAGAATGTTATAGGC-3’ | 5’-GGTGGTGTTGATAAGCAGATTGG-3’ |
| *Pi3k* | 5’-ACACCACGGTTTGGACTATGG-3’ | 5’-GGCTACAGTAGTGGGCTTGG-3’ |
| *Akt1* | 5’-ATGAACGACGTAGCCATTGTG-3’ | 5’-TTGTAGCCAATAAAGGTGCCAT-3’ |
| *Stat3* | 5’-CAATACCATTGACCTGCCGAT-3’ | 5’-GAGCGACTCAAACTGCCCT-3’ |
| *Vegfa* | 5’-CTGCCGTCCGATTGAGACC-3’ | 5’-CCCCTCCTTGTACCACTGTC-3’ |
| *Egfr* | 5’-GCCATCTGGGCCAAAGATACC-3’ | 5’-GTCTTCGCATGAATAGGCCAAT-3’ |

**Supplementary Table S3.** Composition and antioxidant activity of Yinchen Sini Decosion (YCSND).

| **Index** | **Contents** |
| --- | --- |
| DPPH scavenging activity (mg Vc/g) | 73.76 ± 0.56 |
| ∙OH scavenging activity (mg VC/g) | 57.05 ± 0.22 |
| ABTS scavenging activity (mg VC/g) | 15.43 ± 2.03 |
| O_2_^-^ scavenging activity (mg VC/g) | 62.26 ± 3.09 |
| Total Flavonoid (mg RE/g) | 92.70 ± 3.37 |
| Total phenolics (mg GAE/g) | 39.76 ± 0.83 |
| Polysaccharide (mg Glu/g) | 408.55 ± 15.04 |

The results were expressed as the mean ± SD.

**Supplementary Table S4.** Metabolites of Yinchen Sini Decoction (YCSND) by UHPLC-QE-MS.

| **NO** | **t_R_** | **Identification** | **Formula** | **Measured（m/z）** | **Ion mode**  **（m/z）** | **ppm** |
| --- | --- | --- | --- | --- | --- | --- |
| Y1 | 0.28 | Scopoletin | C_10_H_8_O_4_ | 193.0501 | [M+H]^+^ | 0.47 |
| Y2 | 0.81 | Nicotinic acid | C_6_H_5_NO_2_ | 122.0235 | [M-H]^-^ | 4.32 |
| Y3 | 1.16 | Gallic acid | C_7_H_6_O_5_ | 169.0133 | [M-H]^-^ | 1.80 |
| Y4 | 1.19 | Adenine | C_5_H_5_N_5_ | 136.0622 | [M+H]^+^ | 1.12 |
| Y5 | 1.30 | Proline | C_5_H_9_NO_2_ | 114.0548 | [M-H]^-^ | 1.32 |
| Y6 | 1.86 | Isotalatizidine | C_23_H_37_NO_5_ | 408.2749 | [M+H]^+^ | 0.33 |
| Y7 | 3.11 | 5-Hydroxyferulic acid | C_10_H_10_O_5_ | 209.0450 | [M-H]^-^ | 0.17 |
| Y8 | 3.25 | 4-hydroxybenzoic acid | C_7_H_6_O_3_ | 137.0233 | [M-H]^-^ | 2.17 |
| Y9 | 3.34 | Karakoline | C_22_H_35_NO_4_ | 378.2644 | [M+H]^+^ | 1.13 |
| Y10 | 3.35 | Ferulic acid | C_10_H_10_O_4_ | 193.0498 | [M-H]^-^ | 1.19 |
| Y11 | 3.56 | Caffeic acid | C_9_H_8_O_4_ | 181.0500 | [M+H]^+^ | 0.19 |
| Y12 | 3.57 | Cryptochlorogenic acid | C_16_H_18_O_9_ | 355.1027 | [M+H]^+^ | 0.97 |
| Y13 | 3.68 | Bullatine G | C_22_H_31_NO_3_ | 358.2380 | [M+H]^+^ | 0.06 |
| Y14 | 3.68 | Aconine | C_25_H_41_NO_9_ | 500.2863 | [M+H]^+^ | 0.50 |
| Y15 | 3.95 | Sweroside | C_16_H_22_O_9_ | 357.1197 | [M-H]^-^ | 1.88 |
| Y16 | 4.21 | 3-Hydroxybenzaldehyde | C_7_H_6_O_2_ | 121.0282 | [M-H]^-^ | 1.77 |
| Y17 | 4.37 | Fuziline | C_24_H_39_NO_7_ | 454.2797 | [M+H]^+^ | 0.73 |
| Y18 | 4.55 | 1-Caffeoylquinic acid | C_16_H_18_O_9_ | 355.1026 | [M+H]^+^ | 1.12 |
| Y19 | 4.59 | Racanisodamine | C_17_H_23_NO_4_ | 306.1705 | [M+H]^+^ | 1.80 |
| Y20 | 4.60 | Thymol | C_10_H_14_O | 151.1120 | [M+H]^+^ | 0.07 |
| Y21 | 4.66 | Neoline/Bullatine B | C_24_H_39_NO_6_ | 438.2855 | [M+H]^+^ | 1.15 |
| Y22 | 4.78 | Vicenin II | C_27_H_30_O_15_ | 593.1532 | [M-H]^-^ | 2.03 |
| Y23 | 4.81 | Delsoline | C_25_H_41_NO_7_ | 468.2953 | [M+H]^+^ | 1.42 |
| Y24 | 4.84 | Hetisine | C_20_H_27_NO_3_ | 330.2063 | [M+H]^+^ | 2.04 |
| Y25 | 4.89 | 3,5-Dicaffeoylquinic acid | C_25_H_24_O_12_ | 517.1357 | [M+H]^+^ | 1.32 |
| Y26 | 5.06 | Vanillin | C_8_H_8_O_3_ | 153.0549 | [M+H]^+^ | 0.41 |
| Y27 | 5.14 | 3-O-Feruloylquinic acid | C_17_H_20_O_9_ | 367.1033 | [M-H]^-^ | 1.78 |
| Y28 | 5.20 | RUTIN | C_27_H_30_O_16_ | 611.1620 | [M+H]^+^ | 0.02 |
| Y29 | 5.28 | 4-Hydroxycinnamic acid | C_9_H_8_O_3_ | 163.0390 | [M-H]^-^ | 0.25 |
| Y30 | 5.35 | Talatisamine | C_24_H_39_NO_5_ | 422.2907 | [M+H]^+^ | 1.62 |
| Y31 | 5.40 | Harmane | C_12_H_10_N_2_ | 183.0918 | [M+H] ^+^ | 0.86 |
| Y32 | 5.40 | Isoschaftoside | C_26_H_28_O_14_ | 565.1550 | [M+H]^+^ | 1.77 |
| Y33 | 5.64 | 1,3-Dicaffeoylquinic acid | C_25_H_24_O_12_ | 517.1356 | [M+H]^+^ | 1.22 |
| Y34 | 5.76 | Kaempferol | C_15_H_10_O_6_ | 287.0557 | [M+H]^+^ | 2.41 |
| Y35 | 5.90 | Naringenin | C_15_H_12_O_5_ | 273.0758 | [M+H]^+^ | 0.69 |
| Y36 | 5.94 | Isoscopoletin | C_10_H_8_O_4_ | 193.0500 | [M+H]^+^ | 0.16 |
| Y37 | 6.03 | (+)-Cannabidiol | C_21_H_30_O_2_ | 332.2589 | [M+NH_4_]^+^ | 0.30 |
| Y38 | 6.08 | Quercetin-3-O-galactoside | C_21_H_20_O_12_ | 463.0884 | [M-H]^-^ | 1.30 |
| Y39 | 6.12 | Nicotiflorin | C_27_H_30_O_15_ | 595.1654 | [M+H]^+^ | 1.07 |
| Y40 | 6.37 | Quercetin | C_15_H_10_O_7_ | 303.0503 | [M+H]^+^ | 1.11 |
| Y41 | 6.48 | Luteolin | C_15_H_10_O_6_ | 287.0555 | [M+H]^+^ | 1.73 |
| Y42 | 6.48 | Kaempferol-3-O-glucoside | C_21_H_20_O_11_ | 449.1078 | [M+H]^+^ | 2.67 |
| Y43 | 6.53 | 2',4'-dihydroxyacetophenone | C_8_H_8_O_3_ | 151.0389 | [M-H]^-^ | 0.91 |
| Y44 | 6.54 | Quinic acid | C_7_H_12_O_6_ | 191.0550 | [M-H]^-^ | 0.03 |
| Y45 | 6.66 | Salicylic acid | C_7_H_6_O_3_ | 137.0233 | [M-H]^-^ | 2.18 |
| Y46 | 6.67 | Galangin | C_15_H_10_O_5_ | 271.0604 | [M+H]^+^ | 1.43 |
| Y47 | 6.73 | Apigenin-8-C-glucoside | C_21_H_20_O_10_ | 433.1142 | [M+H]^+^ | 0.37 |
| Y48 | 6.85 | Chlorogenic acid | C_16_H_18_O_9_ | 355.1025 | [M+H]^+^ | 1.33 |
| Y49 | 6.92 | Genistein | C_15_H_10_O_5_ | 271.0605 | [M+H]^+^ | 1.69 |
| Y50 | 6.92 | Azelaic acid | C_9_H_16_O_4_ | 189.1125 | [M+H]^+^ | 2.79 |
| Y51 | 7.04 | Isorhamnetin | C_16_H_12_O_7_ | 317.0655 | [M+H]^+^ | 1.67 |
| Y52 | 7.04 | Scoparone | C_11_H_10_O_4_ | 207.0656 | [M+H]^+^ | 2.85 |
| Y53 | 7.38 | Isoliquiritin | C_21_H_22_O_9_ | 417.1201 | [M-H]^-^ | 0.18 |
| Y54 | 7.52 | Licochalcone B | C_16_H_14_O_5_ | 285.0768 | [M-H]^-^ | 0.83 |
| Y55 | 7.53 | Chrysin | C_15_H_10_O_4_ | 253.0510 | [M-H]^-^ | 0.14 |
| Y56 | 7.67 | Liquiritigenin | C_15_H_12_O_4_ | 257.0806 | [M+H]^+^ | 1.48 |
| Y57 | 7.67 | Mesaconitine | C_33_H_45_NO_11_ | 632.3066 | [M+H]^+^ | 0.58 |
| Y58 | 7.72 | Ononin | C_22_H_22_O_9_ | 431.1342 | [M+H]^+^ | 0.53 |
| Y59 | 7.80 | Benzoylhypaconine | C_31_H_43_NO_9_ | 574.3013 | [M+H]^+^ | 0.45 |
| Y60 | 7.84 | Camphor | C_10_H_16_O | 153.1275 | [M+H]^+^ | 3.53 |
| Y61 | 8.01 | 7-Methoxycoumarin | C_10_H_8_O_3_ | 177.0547 | [M+H]^+^ | 1.52 |
| Y62 | 8.03 | Glabrolide | C_30_H_44_O_4_ | 469.3325 | [M+H]^+^ | 1.04 |
| Y63 | 8.15 | Isochlorogenic acid B | C_25_H_24_O_12_ | 517.1352 | [M+H]^+^ | 0.35 |
| Y64 | 8.25 | Licoricesaponin G2 | C_42_H_62_O_17_ | 839.4056 | [M+H]^+^ | 0.48 |
| Y65 | 8.33 | Carveol | C_10_H_16_O | 135.1169 | [M+H]^+^ | 0.93 |
| Y66 | 8.35 | Dihydrocapsaicin | C_18_H_29_NO_3_ | 308.2226 | [M+H]^+^ | 1.85 |
| Y67 | 8.74 | Naringenin chalcone | C_15_H_12_O_5_ | 271.0616 | [M-H]^-^ | 2.13 |
| Y68 | 8.97 | Isoliquiritigenin | C_15_H_12_O_4_ | 257.0807 | [M+H]^+^ | 1.27 |
| Y69 | 9.26 | Ursolic acid | C_30_H_48_O_3_ | 439.3578 | [M+H]^+^ | 0.56 |
| Y70 | 9.31 | Hypaconitine | C_33_H_45_NO_10_ | 616.3109 | [M+H]^+^ | 0.17 |
| Y71 | 10.46 | Glycyrrhizic acid | C_42_H_62_O_16_ | 823.4109 | [M+H]^+^ | 0.09 |
| Y72 | 10.50 | Dihydroartemisinic acid | C_15_H_24_O_2_ | 237.1852 | [M+H]+ | 0.73 |
| Y73 | 10.56 | Artemisinic acid | C_15_H_22_O_2_ | 235.1696 | [M+H]+ | 1.83 |
| Y74 | 10.76 | Curcumin | C_21_H_20_O_6_ | 369.1335 | [M+H]+ | 1.46 |
| Y75 | 10.96 | 6-Gingerol | C_17_H_26_O_4_ | 293.1764 | [M-H]^-^ | 1.42 |
| Y76 | 11.00 | Germacrone | C_15_H_22_O | 219.1747 | [M+H]+ | 1.23 |
| Y77 | 11.46 | Licochalcone A | C_21_H_22_O_4_ | 337.1445 | [M-H]^-^ | 1.39 |
| Y78 | 11.48 | Phenylacetaldehyde | C_8_H_8_O | 121.0652 | [M+H]+ | 1.53 |
| Y79 | 12.23 | 2-Hydroxyacetophenone | C_8_H_8_O_2_ | 137.0600 | [M+H]+ | 0.19 |
| Y80 | 12.33 | Ligustilide | C_12_H_14_O_2_ | 191.1071 | [M+H]+ | 0.26 |
| Y81 | 12.34 | 6-Shogaol | C_17_H_24_O_3_ | 277.1803 | [M+H]+ | 1.05 |
| Y82 | 13.65 | 18 beta-Glycyrrhetintic Acid | C_30_H_46_O_4_ | 469.3323 | [M-H]^-^ | 1.57 |
| Y83 | 13.80 | Di-n-butyl phthalate | C_16_H_22_O_4_ | 279.1594 | [M+H]+ | 1.39 |
| Y84 | 13.99 | 8-Shogaol | C_19_H_28_O_3_ | 305.2118 | [M+H]+ | 2.65 |
| Y85 | 14.76 | Morusin | C_25_H_24_O_6_ | 419.1511 | [M-H]^-^ | 0.28 |
| Y86 | 15.30 | Betulinic acid | C_30_H_48_O_3_ | 455.3535 | [M-H]^-^ | 1.08 |
| Y87 | 27.94 | Phenylacetic acid | C_8_H_8_O_2_ | 135.0441 | [M-H]^-^ | 0.63 |
| Y88 | 28.50 | Pyrogallol | C_6_H_6_O_3_ | 125.0233 | [M-H]^-^ | 2.06 |
| Y89 | 28.71 | 18Alpha-Glycyrrhetinic Acid | C_30_H_46_O_4_ | 471.3482 | [M+H]^+^ | 0.36 |


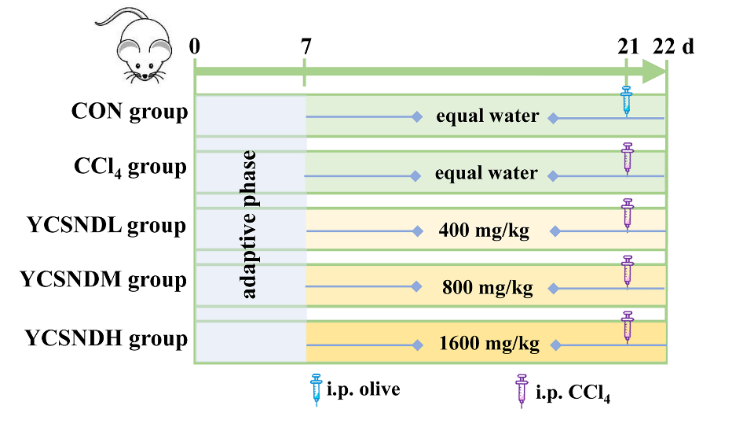


**Supplementary Figure 1.** The flowchart of the animal experiment.


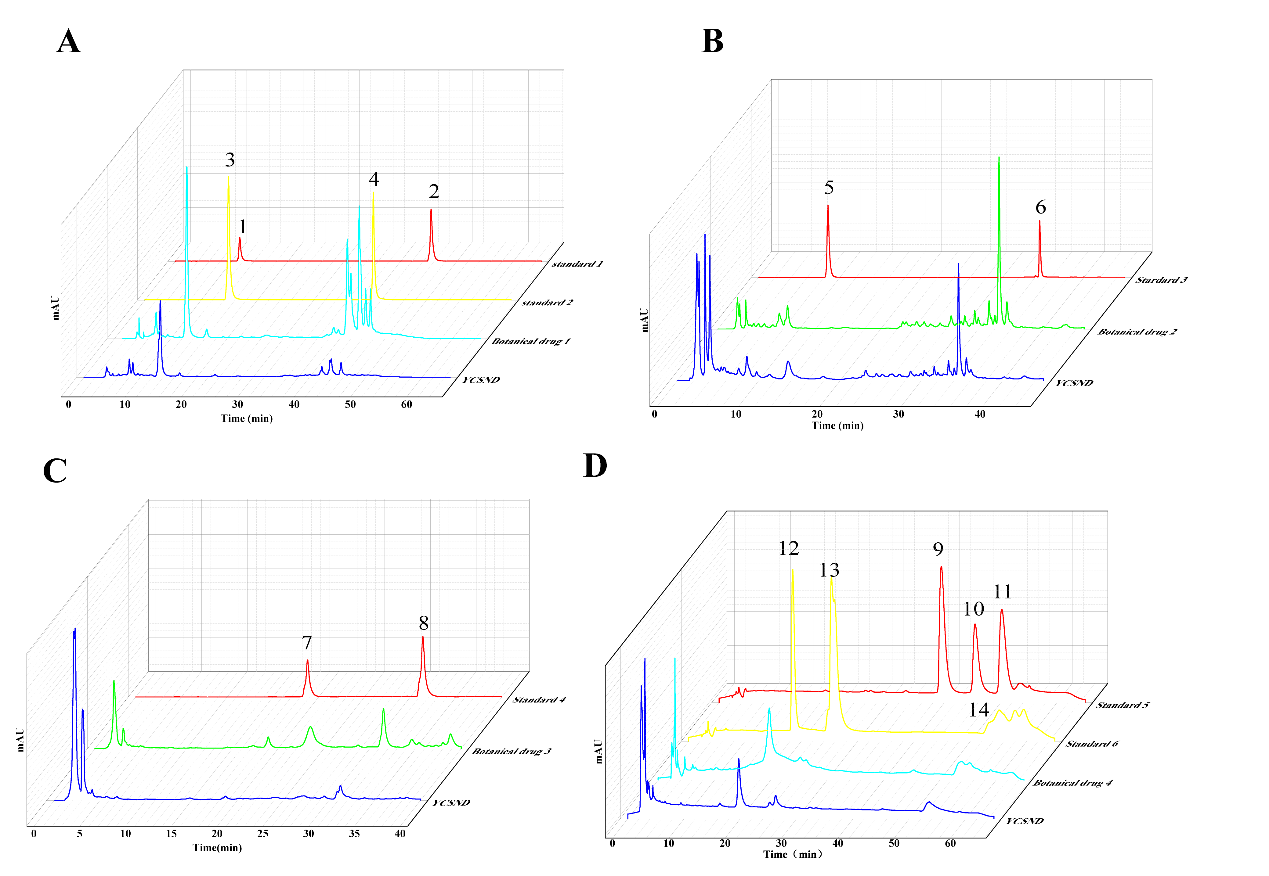


**Supplementary Figure 2.** The fingerprints of Yinchen Sini Decoction (YCSND) and the quality control of its four botanical drugs. (A)-(D) showed YCSND and its botanical drugs fingerprints in the different conditions from program 1-4 by HPLC. Peaks: 1, [chlorogenic acid](javascript:;); 2, isochlorogenic acid; B; 3, caffeic acid; 4, scoparone; 5, liquiritin; 6, glycyrrhizic acid; 7, 6-gingerol; 8, 6-shogaol; 9, mesaconitine; 10, [hypaconitine](javascript:;); 11, aconitine; 12, benzoylneaconitine; 13, benzoylaconitine; 14, benzoylhypaconitine. Sample: Biotanical drug 1, *Artemisia capillaris* Thunb.; Biotanical drug 2, honey-fried *Glycyrrhiza glabra L*.; Botanical drug 3, *Zingiber officinale* Roscoe; Botanical drug 4, *Aconitum carmichaelii* Debeaux.


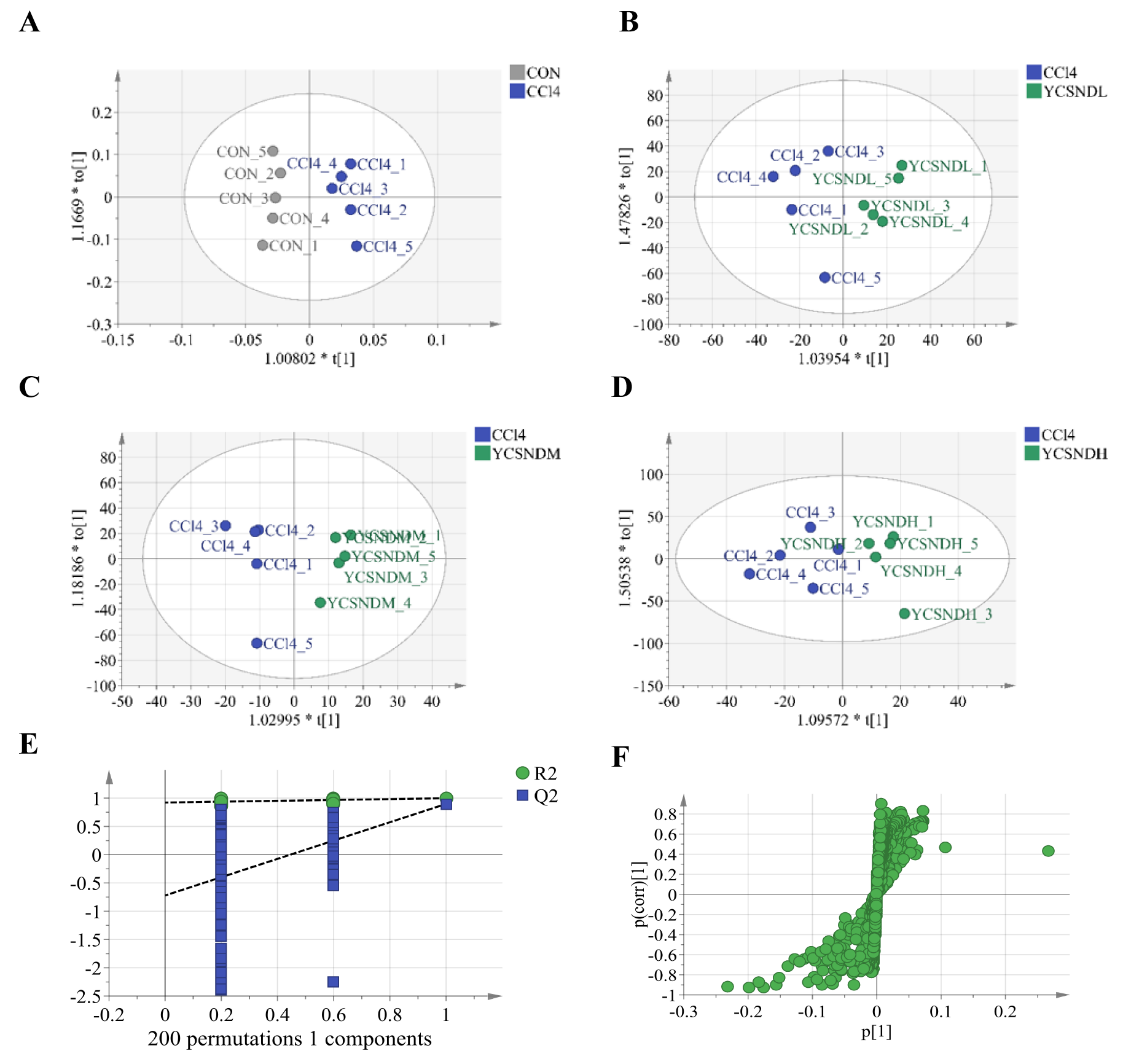


**Supplementary Figure 3.** Multivariate data analysis from ^1^H-NMR. (A) OPLS-DA score plots from the CON and CCl_4_ groups. (B) OPLS-DA score plots from the CCl_4_ and YCSNDL groups. (C) OPLS-DA score plots from the CCl_4_ and YCSNDM groups. (D) OPLS-DA score plots from the CCl_4_ and YCSNDH groups. (E) Permutation test (n=200 times) was used to validate the OPLS-DA model. (F) S-plot in CON and CCl_4_ groups.


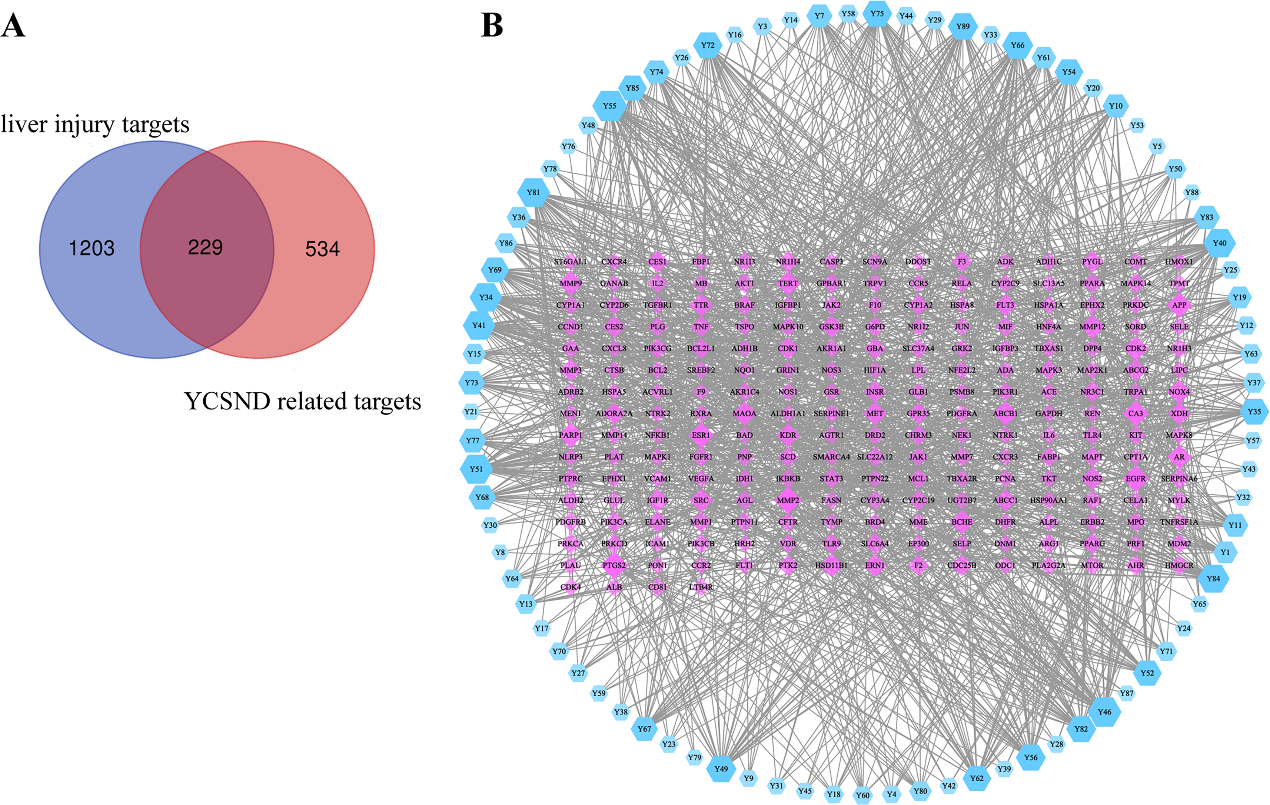


**Supplementary Figure 4.** Network analysis of Yinchen sini decoction (YCSND) in the treatment of acute liver injury (ALI). (A) Overlapping gene symbols between ALI and YCSND. (B) Metabolite-Targets network. The blue node represents metabolites and the pink node represents gene targets.


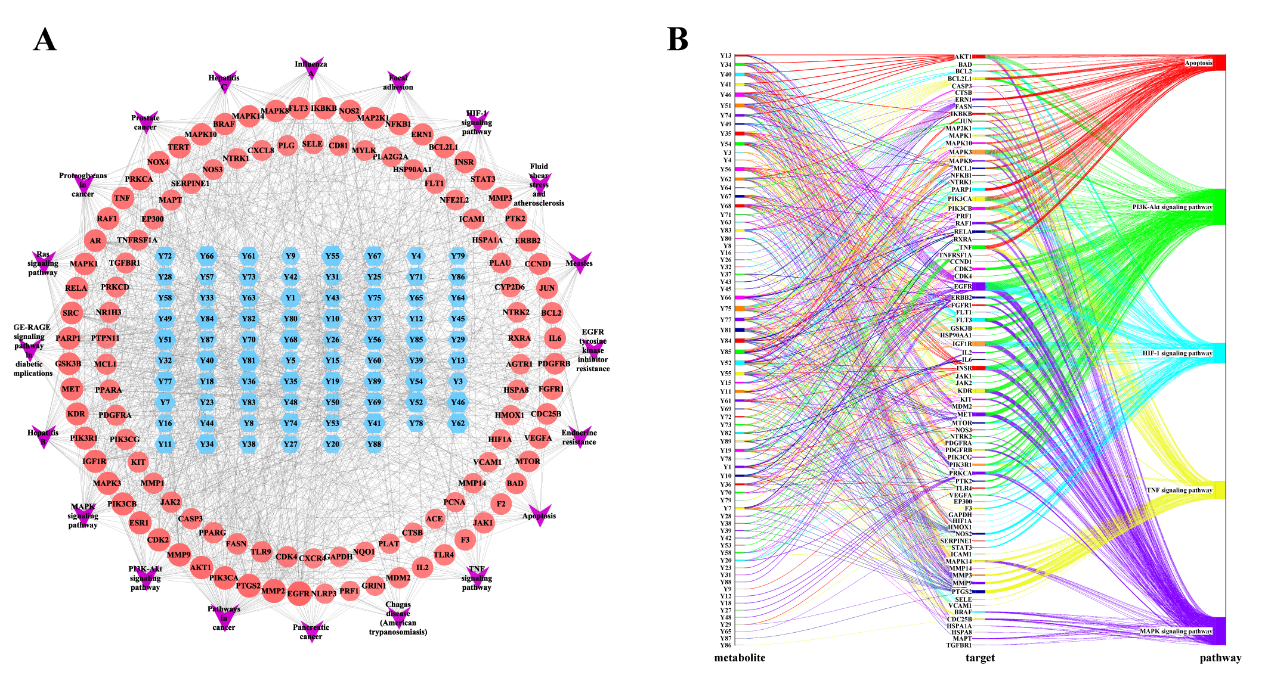


**Supplementary Figure 5.** Metabolite-target-pathway (M-T-P) network of YCSND against liver injury. (A) The network of compounds, targets and the 20 KEGG pathways. Red nodes represent targets, blue nodes represent metabolites of YCSND and purple nodes represent pathways. (B) M-T-P network of five key KEGG pathways and related targets and metabolites.


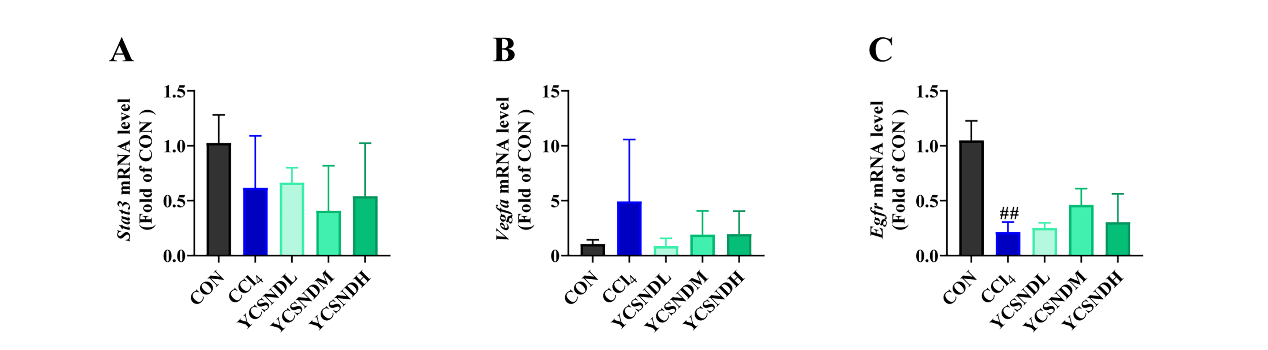


**Supplementary Figure 6.** The results of experimental verification in *vivo.* (A-I) The mRNA level of *Egfr*, *Stat3* and *Vegfa* of CCl_4_-induced ALI in mice by YCSND treatment respectively. Data are showed as mean ± SD. #*p* < 0.05, ##*p* < 0.01, ###*p* < 0.001 vs. CON group; **p* < 0.05, ***p* < 0.01, ****p* < 0.001 vs. CCl_4_ group.
